# Modernization of Control of Pathogenic Micro-Organisms in the Food-Chain Requires a Durable Role for Immunoaffinity-Based Detection Methodology—A Review

**DOI:** 10.3390/foods10040832

**Published:** 2021-04-11

**Authors:** Aldert A. Bergwerff, Sylvia B. Debast

**Affiliations:** Laboratory of Clinical Microbiology and Infectious Diseases, Isala Hospital, Dr. van Heesweg 2, NL-8025 AB Zwolle, The Netherlands; s.b.debast@isala.nl

**Keywords:** food microbiology, immunoaffinity assays, immunoagglutination, immunosensors, immunochromatographic testing, immunomagnetic separation, one health, pathogenic micro-organisms, responsive monitoring, review

## Abstract

Food microbiology is deluged by a vastly growing plethora of analytical methods. This review endeavors to color the context into which methodology has to fit and underlines the importance of sampling and sample treatment. The context is that the highest risk of food contamination is through the animal and human fecal route with a majority of foodborne infections originating from sources in mass and domestic kitchens at the end of the food-chain. Containment requires easy-to-use, failsafe, single-use tests giving an overall risk score in situ. Conversely, progressive food-safety systems are relying increasingly on early assessment of batches and groups involving risk-based sampling, monitoring environment and herd/flock health status, and (historic) food-chain information. Accordingly, responsible field laboratories prefer specificity, multi-analyte, and high-throughput procedures. Under certain etiological and epidemiological circumstances, indirect antigen immunoaffinity assays outperform the diagnostic sensitivity and diagnostic specificity of e.g., nucleic acid sequence-based assays. The current bulk of testing involves therefore *ante*- and *post-mortem* probing of humoral response to several pathogens. In this review, the inclusion of immunoglobulins against additional invasive micro-organisms indicating the level of hygiene and *ergo* public health risks in tests is advocated. Immunomagnetic separation, immunochromatography, immunosensor, microsphere array, lab-on-a-chip/disc platforms increasingly in combination with nanotechnologies, are discussed. The heuristic development of portable and ambulant microfluidic devices is intriguing and promising. *Tant pis*, many new platforms seem unattainable as the industry standard. Comparability of results with those of reference methods hinders the implementation of new technologies. Whatever the scientific and technological excellence and incentives, the decision-maker determines this implementation after weighing mainly costs and business risks.

## 1. Introduction

The ever-increasing number of studies on the determination of micro-organisms (further abbreviated as MOs) gives the impression that the development of analytical platforms is solely technology-driven. Many of these developments seem not to connect to or seem not to fit in the daily practice of systems that should warrant the safety of food. Several inventions stick in testing relative pure reference materials or test spiked field samples only. Such samples do not reflect naturally infected animals, plants, or food products.

This paper will discuss which categories of disease-causing MOs need most and immediate attention and control (Section 1.1 and Section 1.2). Examination of failing hygiene, pest control, and extrinsic and extramural factors will clarify the need for surveillance of the environment and rationalize the type of testing needed, *viz*. tests delivering multiple data from a series of samples timely and accurately (Section 1.3). Subsequently, this paper will show that consumers and persons preparing meals are responsible for the majority of foodborne infections and wonders whether there is a more prominent role for tests in the kitchen and domestic situations (Section 1.3). To understand how immunoaffinity (IA) testing may fit in all of this, in particular in intervention and control programs, this review presents first the basic concepts and historical perspectives of MOs analysis (Section 2.2 and Section 2.3).

The conventional, emerging, and novel alternative approaches lined up in this paper are compared for their tradeoffs, merits, demerits, and usefulness in the light of a demand for more confidence (Section 2.6, Section 2.7 and Section 3). The authors recognize that their views are based upon Western standards with the comfort of advanced technology at one’s disposal. Colleagues from other regions may have other opinions on the utility of techniques and validity of some conclusions.

The authors realize also the numerous reviews and overviews which appeared on the subject. They attempted to look at developments in food microbiology analysis in another way by combining views on monitoring, reliability of results, the efficacy of the intervention, etc. It is for this reason that an example of *Salmonella* intervention in pig production is presented as a showcase of the analytical challenges in practice (Section 4).

This review does not desire to discuss specific technical and (bio)chemical details of innovative IA platforms. The reader interested in these details is given many (recent) references and other starting points to find this information. In addition, the article is written with routine, high-volume analyses of the food-chain in mind. Nevertheless, the authors are also familiar with specialistic methodology to determine bioagents that are non-cultivable or fastidious to determine. These methods are commonly Herculean for screening purposes and are therefore not discussed or only touched. Finally, the paper will elaborate modestly on the future of IA-based platforms, methodology, and the microbes of concern (Section 5 and Section 6). After all, the SARS-CoV-2 pandemic has made us frustratingly and unmistakably clear: not macro-organisms but micro-organisms rule the world!

### 1.1. Food and Micro-Organisms

Eating and digesting food will maximize the entropy of the consumed plant or animal [1,2]. Food stabilizes the entropy of the consumer and facilitates the renewal of life. Food is thus essential to life but comes with the risk of other forms of entropy maximization. For humans, these (physical injury) risks do not arise from catching, harvesting, processing, or preparing food *per se*. Generally, food contains invisible micro-organisms; a term suggesting tiny living material with an autonomous nature. It can be debated whether this is completely true. As defined by the European Commission, MOs compromise bacteria, viruses, yeasts, molds, algae, parasitic protozoa, microscopic parasitic helminths, and their toxins and metabolites [3]. So, not all MOs are living materials and autonomous.

MOs are part of biogeochemical cycles, are highly adaptable, can survive anywhere and in all facets of life. Most MOs are “friendly” and many have closed a pact with plants, animals, and humans. In particular symbiotic bacteria protect and support our skin and gut, and help to convert certain food components while also producing e.g., vitamin K. In reality, microbes in the gut are essential for a healthy person stimulating the immune system. Beneficial MOs ferment (unpalatable) matters into favorable and enjoyable food products. Besides adding taste and quality, MOs can spoil food products and/or form a health threat for food workers and consumers. Following the invasion of a host, some MOs can replicate and cause infectious diseases. Pathogenic MOs can be harmful even without invasion of the eater by leaving toxins in the food and causing so-called toxic infections. In other cases, not only the injurious action of the MO but also the consumer’s immune response to the MO leads to health damage. For example, although rare and affecting only approximately in 1 out of 1,000 infected individuals, different reactive (autoimmune) diseases are possible consequences of a *Campylobacter* infection [4,5]. Annually, 1,400 cases of reactive arthritis, 60 cases of Guillain-Barré syndrome, and 10 cases of inflammatory bowel disease as a result of campylobacteriosis are estimated in The Netherlands alone [6]. Here, *Campylobacter* is an example of bacterial zoonosis, i.e., a pathogen transmitted from animal to human.

Microbial hazards, like zoonoses, can enter the food-chain at any point: water, soil, in the growing, weaning, fattening phase up to the preparation of a meal. Water and soil are listed not without reason. They may contain harmful bacteria, parasites, and viruses which infect or contaminate plant or animal. For example, cows may contract zoonotic cysticercosis from feeding on tainted grass. Pet food is a part of that food-chain as well and also requires high hygienic standards. In several surveys, disease-causing agents, such as *Salmonella*, *Listeria*, toxin-producing *Escherichia coli*, *Mycobacterium* spp., *Sarcocystis* spp. and many other parasites were found in, predominantly raw, cat and dog food, not only diseasing companion animals but also transmitting the pathogens to (juvenile) humans [7,8,9]. Besides the indirect route, remind that pet food is consumed occasionally by people who cannot afford other food [10].

A subject often forgotten, is that food safety does not only concern the final consumer, but also the professionals coming into contact with infected or contaminated animals, plants, and food products, namely the butcher, (spouse and children of) farmers, fishermen, inspector, processor, retailer, slaughterer, veterinarian, etc. [11], and residents close to farms and slaughterhouses. Many risks, such as hepatitis E virus (HEV), *Salmonella*, *Streptococcus suis*, *Vibrio* sp. (fish products), have been underestimated and underreported for food-workers for a long time [12,13]. As an inquisitiveness, *vice versa*, professionals can infect plants (hepatitis A virus (HAV)), animals (*Streptococcus* Lancefield, *Taenia saginata* or *T. solium*), also referred to as anthropozoonosis, or food products *(Cryptospora*, *Cyclospora, Giarda*, HAV, norovirus, *Salmonella*, *Streptococcus aureus*, *Shigella*) as well.

### 1.2. Need to Contain Foodborne Pathogenic Micro-Organisms

Food contaminated with chemical substances and harmful MOs cause over 200 diseases [14]. An estimated number of 600 million persons worldwide fall ill from foodborne infections every year [14]. A total of 420,000 fatalities and 33 million disability-adjusted life years (DALYs), i.e., lost healthy years, are ascribed to food-transmitted diseases. Others report almost one million deaths due to water- and foodborne gastroenteritis by thirteen MOs in children aged less than five years in Africa, Asia, and Latin America [15]. When only zoonotic risks of food are considered, the most commonly detected causative agents in 2017 in Europe were bacteria (33.9%) followed by bacterial toxins (17.7%), viruses (9.8%), other causative agents (2.2%), and parasites (0.4%) [16]. The foods involved were eggs (23.0%), poultry meat (18.5%), fishery products including crustaceans, bivalves (22.4%), meat products other than poultry (21.7%), and dairy (14.4%) [16].

#### 1.2.1. Bacteria

Most bacterial foodborne illnesses worldwide are caused by *Campylobacter*, namely an estimated 96 million cases in 2010 [17]. However, on a world-scale, the highest health burden of foodborne MOs comes from non-typhoidal *Salmonella enterica* infections, namely 4.07 million DALYs in 2010 [17]. In most confirmed cases in EU member states and associated countries, food was the carrier of pathogens with a zoonotic origin (Table 1) [18,19]. Despite its much lower incidence, the highest case fatality was caused by *Listeria* (17.6%), whereas the top two, campylobacteriosis and salmonellosis, caused 0.03% and 0.22% mortality, respectively [19].

**Figure 1:**
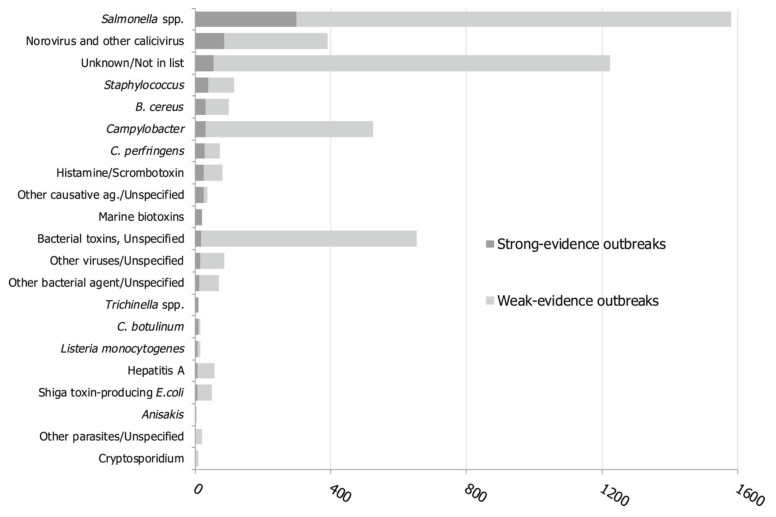
Food and water-borne outbreaks, i.e., cases in which ≥2 persons fell ill from the same food and the same agent, per causative pathogen in 2018 in the EU and associated countries. The explanation of “other” relevant for this paper is as follows. Other bacteria include *Aeromonas hydrophila*, (enteroinvasive (EIEC) or enterotoxigenic (ETEC)) *Escherichia coli*, *Enterococcus*, *Leptospira* spp., *Shigella* spp., *Shigella flexneri*, *Shigella sonnei*, *Yersinia enterocolitica*, and unspecified bacteria. Other viruses include adenovirus, flavivirus, hepatitis E virus, rotavirus, and unspecified viruses. Other parasites include *Giardia intestinalis* (*lamblia*), *Giardia* spp., *Taenia saginata*, and unspecified parasites. Figure courtesy of EFSA from [18].

When studying food-caused outbreaks exclusively, *viz*. cases in which two or more persons fall ill from the same foodborne sickness after eating or drinking the same food, *Salmonella* is causing substantially more instances than *Campylobacter* (Figure 1). It seems that certain strains, such as *Salmonella enterica* Enteritidis, evolved in such a way that it efficiently transmits to humans [20]. Although their incidence is low, opportunistic bacterial contaminants are causing, some very serious, hospitalizations, such as *Enterobacter sakazakii, Klebsiella* spp., *Citrobacter* spp., *Serratia* spp. [21,22].

#### 1.2.2. Parasites

From Figure 1 it is evident that bacteria play an important role, but that there are microbial pathogens from other phylogenic domains to reckon with. Besides biotoxins, such as histamine and phycotoxins, viruses and parasites should not be neglected. Parasites fall into two main groups: protozoa and helminths (worms) and they are characterized by, sometimes very complicated, life cycles which can take years to complete. The fecal-oral route is predominantly contaminating the food-chain. Parasitic eggs, oocytes, of some species can easily survive years in the environment. Fruits or salads tainted by contaminated water may be the source of an infection among which e.g., tapeworm *Echinococcus multilocularis* and *Echinococcus granulosis* are feared. Large outbreaks through insufficiently decontaminated surface water prepared for drinking water is frequently the cause of human protozoan infections in the Eastern-Europe, UK, and the USA, such as *Giardia*, *Cryptosporidium*, and *Cyclospora* [23].

The potential implications of foodborne worms and protozoans, such as *Anisakis* spp., *Taenia* spp., *Sarcocystis* (*sui*) *hominis*, *Toxoplasma gondii*, or *Trichinella* spp., should not be underestimated. To demonstrate the impact of one of these parasites, approximately 45 million persons worldwide, of which 11 million persons in Europe, suffer from taeniosis caused by *Taenia saginata* [24]. A *T. saginata*, or beef tapeworm, infection affects annually up to 30,000 persons in The Netherlands alone [23]. This mild infection runs exclusively through cattle with humans as the final host. For cattle to become infected it has to come in contact with human sewage. As a consequence, prevalence is higher in countries with poor sewage control [25]. Taeniosis is thus also a sign of other pathogenic risks associated with improper hygiene. The infection is one of the blind spots in our surveillance systems; the sensitivity of the obligatory visual inspection for the causative worm is only 10% to 30% [26]. There is thus a world to win for test developers, routine test laboratories, and authorities. An available IA method is used for research purposes only and not suitable for routine analyses [27].

#### 1.2.3. Viruses

Nearly all food-transmitted viruses originate from the human gut. Infections are predominantly caused by fecal contamination of (prepared) food as a result of insufficient personal hygiene of people handling the food. An important risk factor is the pre-symptomatic spreading of a virus by an e.g., infected cook or worker in the food industry. Consequently, several important health-threatening virions become foodborne at the end of the food-chain. Potential food-contaminating human fecal viruses belong to the adenoviruses, astroviruses, caliciviruses (norovirus), enteroviruses, hepatoviruses, parvoviruses, and rotaviruses. In this list is the all-time highest-scoring food-poisoning organism worldwide causing an estimated 125 million cases of gastro-enteritis in 2010: norovirus [17]. To make clear that norovirus is not an issue of developing regions alone, 60% and 75% of all foodborne illnesses in the European region [28] and in the USA, respectively, are caused by norovirus. Norovirus is highly contagious: the infectious dose can be only a few virus particles [29].

An indirect human fecal-to-food route is the contamination of bivalves cultured in estuaries, coastal regions, or other places with (brackish) water tainted by human excreta. In these cases, sewage can come from inefficiently working water purification systems dumping its pathogen-containing product in open water systems. Not to underestimate are also the sources of (a)symptomatic carriers defecating in open waters and fields of which many cases have been described in developed countries. In this way, bivalves were a vehicle for poliovirus, HAV, HEV, norovirus (and several dangerous bacteria such as *Shigella*, *Vibrio cholera*) [30]. Only 10–100 HAV particles are sufficient to infect a person and similar to norovirus, it is predominantly poor hygiene that the virus is transferred via fruit and (undercooked) meat. Fortunately, in none of the 1248 samples of fruit, bivalves and meat HAV were detected in the EU in 2018 [18]. Concordantly, none of the 535 fruit and vegetable samples were positive in 2019 either [19].

An exception to the human fecal route, although its etiology and epidemiology are not yet completely clear, is the risk of an HEV infection through consumption of raw or mildly prepared pig, wild boar, and deer meat products [31]. Fermented/dried sausages provide the highest HEV risk following raw *porcine* liver. The cause is liver-contaminated diaphragm muscle used to produce sausages.

#### 1.2.4. Other Types of Pathogens

Among bacteria, viruses, parasites, the reader may miss foodborne infections caused by algae, fungi, yeasts, and infectious proteins (prions). The last category does not initiate (adaptive) immune responses and although detectable using IA techniques (e.g., immuno-PCR [32]), it is considered to be beyond the subject of this paper. Whereas immunocompromised patients have to be careful, foodborne algae, fungi, yeasts are seldomly harmful to healthy people. Although these infections are rare, toxicoinfections through the production of, some extremely harmful, toxins by fungi growing in/on plants or (raw) food products are not rare at all [33]. Toxicoinfections with a bacterial origin are caused by food-transmitted toxins produced by for example *Bacillus cereus*, *Clostridium botulinum*, *Clostridium perfringens*, and *Staphylococcus aureus* [34]. Their exercised menace is responsible for 16% of all food- and water-borne outbreaks in the EU in 2017 [18]. Immunoaffinity testing methodology to assess these biotoxin-caused risks is beyond the scope of this review and the subject is only touched.

### 1.3. Failing Containment of Pathogenic Micro-Organisms

With appropriate measures and intervention, the risks of MOs in food can be mitigated greatly if not completely. Their chance of survival in the chain is determined by extrinsic (such as humidity, temperature, gas composition), intrinsic (such as water activity, pH, structure), and implicit (such as the ability of the MO to adapt to its environment) factors which can be controlled. Humankind has discovered and invented many food handling and preparation methods, including cooling, salting, fermentation, drying, acidifying, marinating, to influence the risk of MOs. In some cases, new microbial risks were however introduced unintentionally by these methods, such as the very dreaded botulinum toxin-producing *Clostridium botulinum* bacteria in e.g., air-dried sausage (*botulus* in Latin), dried fish or oil-submerged protein-rich food products (like mushrooms) (see ProMed mailings for case descriptions [35]).

Even when the cooling chain is closed and maintained at low temperatures as a common and effective intervention method should, bacteria, like pathogenic *Yesinia enterocolitica* and *Listeria monocytogenes*, can still grow to risky amounts. Actually, it is hypothesized that *Listeria monocytogenes* has adapted itself to processing plant conditions, such as osmotic, detergent, acid, and oxidative stress [36], and became psychrotrophic, i.e., growing even at low temperatures [37], since the introduction of food-processing plants and home refrigerators, respectively [38].

Under optimal conditions, bacteria can propagate very fast. For example, *Clostridium perfringens* can produce a new generation every ten minutes. MOs can be contained in food by obeying strict (environmental) hygiene and by maintaining a closed cooling chain at low temperatures. Apparently, as a relative number of cases are dropping over the years [39,40], humankind succeeds increasingly well, but, as entropy strives for maximization [2], hygiene and other measures can never be neglected and the risk will never be zero. Therefore, continuous intervention is crucial and food microbiology plays an important role in control.

Notably, over 50% of the registered foodborne infections in Europe and North America were contracted at home [41]. Following domestic causes, food from mass catering, such as fast-food outlets, in-flight caterings, hotels, hospital restaurants, takeaways, causes most salmonellosis cases [39]. Persons preparing food make many food-handling errors, including inadequate handwashing, poor surface cleaning, undercooking, etc. [42]. Knowledge of safe food storage and handling is poorly developed under young consumers [43]. Household sources of infection are mainly contaminated food and water, people, pests, and pets greatly by a fecal-oral route. The reader notices that the bioagents causing a large part of the foodborne illnesses are thus not originating from the original food product upstream in the chain.

Sensible domestic hygiene principles seem partly lost. We learn about correct and incorrect hygiene in the kitchen effectively from watching cooking shows [44]. When asked, people generally are not aware to prepare their salad and vegetables before that of their meat, are not conscious of knife hygiene, hand washing, or refrigerator cleanliness (authors’ outcomes of inquiries, unpublished). It is therefore not a surprise that fecal coliforms originating from intestines of warm-blooded animals, including from ourselves, and a warning for the possible presence of pathogens, were found in all 250 sampled home kitchens [41]. Within the scope of this review, affordable, convenient and reliable microbiological assays for household testing to give a consumer a means to see imperfect cleaning and risks lurking, are hardly available.

The way we prepare food has evolved hugely. A trend is the increasing use of locally produced products that may evade (not maliciously *per se*) usual food-safety inspection regimes. In addition, we seem to appreciate serving raw or barely cooked dishes to emphasize the freshness and excellence of the ingredients used. Along with tidbits of for example uncooked salmon tartare, raw milk directly from the dairy farm, cheese from non-pasteurized milk, and thin slices of beef carpaccio, so fresh are also the parasites, bacteria, and viruses that can reside in/on them. We also increasingly prefer ready-to-eat food and expect that these are pathogen-free. Well, a third of all strong-evidence outbreaks involved buffet meals, mixed food, and unspecified foods [16,39].

## 2. Analytical Microbiology

There is no future without a past. This chapter will therefore first clarify why analysis in the food-chain is indispensable (Section 2.1). Following a short description of the discovery of bacteria, parasites, and viruses (Section 2.2) and their biochemical characteristics (Section 2.3.1), this chapter will throw a light on two important available measuring principles (Section 2.3.2) and suitable sample types for these measuring principles. These insights will help to explain where, what, when, for which purpose, and at what costs measurements are performed (Section 2.4 and Section 2.5) before specific analysis immuno-affinity assay techniques (Section 2.6) are described. This chapter will close with a short description of a few important analysis principles other than those based on a specific reaction of an immunoglobulin with an antigen and their comparison with immunoaffinity assays (Section 2.7).

### 2.1. Why Need to Measure?

Pathogenic MOs in the food-chain are not only a threat to human health, and cause of DALYs and lost lives, they have a socio-economic impact and influence world history [45,46]. The economic losses due to lost labor capacity, health-care costs, damage to product brand, producer’s standing, etc. can be devastating. Although foodborne illnesses are known for thousands of years [47], foodborne infections were considered long not life-threatening. Some pathogens were considered a problem of for example canners, such as botulism, while eggs were regarded sterile as long as the shell was closed [48]. The first serious consumer concern with respect to foodborne pathogens with a significant societal impact was at the end of the 1980s of the last century. These public concerns on infections contracted from contaminated food were surprisingly much later than public concerns on chemical contaminants in food, such as anabolic hormones in particular diethylstilbestrol (DES) [49]. Still, the consumer appears to accept pathogens in food more than regulated and tolerated chemical food contaminants, such as residues of veterinary pharmaceuticals. Nonetheless, in comparison, morbidity and mortality clearly comes prevalently from foodborne MOs, including bacterial and fungal toxins, not from chemical contaminants and additives in food. Colleagues and authors assume chemophobia stronger than fear for ‘natural’ biological phenomena like pathogens, but, apparently, this has not been investigated scientifically [50].

The first big food-scare involving pathogens playing havoc with the food-production industry was namely in 1988 and went into history as the “egg affair” [46]. It started with the impromptu announcement in the UK that salmonellosis in humans was linked with the consumption of *Salmonella*-contaminated eggs and poultry. This declaration was hyped by the press and caused a collapse of the British egg industry. It marked the birth of (supra)national *Salmonella* screening and control programs by organized private and by governmental/NGO parties.

**Figure 2:**
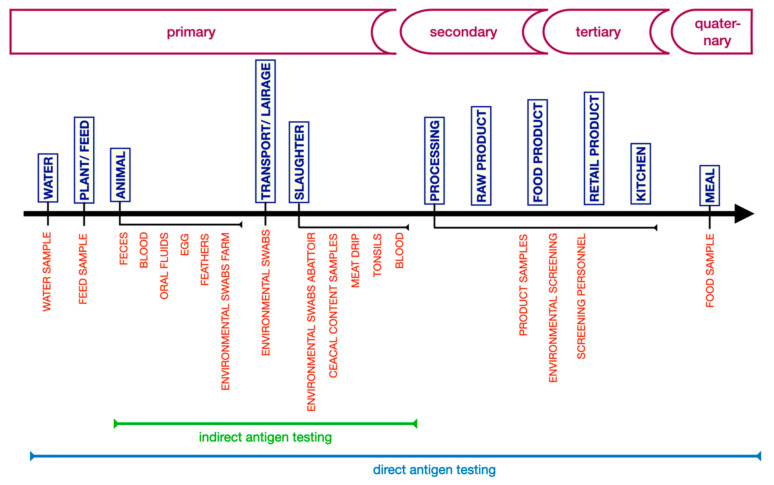
When, what, and how to screen in the animal food production chain (dark blue color) to secure food safety with respect to microbial hazards. Hazard critical point analysis will reveal optimal sampling moments. The sectors (purple color) which determine the type of sampling (red color) and testing (green and light blue colors) possible are indicated. The primary sector includes the pre-harvest stage until slaughter and comprises reproduction, egg and milk, fattening, transport, and slaughter phases. The secondary sector includes all food-processing steps converting milk, eggs, and meat into complex products. The tertiary and quaternary sectors include wholesale, street vendors, catering, institutional kitchens, and finally private kitchens and (domestic) consumption.

Hygiene and control measures are important to stop a bioagent in the food-chain and to prevent harm to the consumer (Figure 2). Whether these general actions are sufficient, and whether additional, specific interventions are necessary, is revealed by continuous, appropriate, and efficacious analysis of (indicators of) bioagents at critical moments in the chain.

### 2.2. Historical Overview of the Discovery of Micro-Organisms

In the awareness of infectious diseases transmitted through food, there is since the discovery of MOs an increasing need to escape agnostic situations and to pin and destroy harmful bioagents in their habitats. However, it took almost two centuries after Antoni van Leeuwenhoek (1632–1723) observed bacteria in 1675 as the first human till Rudolf Virchow (1821–1902) formulated his cellular pathology concept in 1858. Virchow formulated the third dictum of cell theory *Omnis cellula e cellula* (“All cells come from cells”) and coined the term “zoonosis” as he had noticed a link of diseases between man and animals. The new cellular pathogen concept formed the basis of modern (analytical) bacteriology funded by Louis Pasteur (1822–1895) and Robert Koch (1843–1910). In addition, August Gärtner (1848–1934) showed that several bacteria were able to produce thermostable toxins giving cholera and typhus-like diseases after consumption of contaminated food. This finding showed that bacteriology does not end with the absence of a pathogenic bacterium and that food is an important vehicle for diseases.

In parallel, the propagation cycles of several parasites were elucidated mid-19th century and paved the way to modern parasitology. In addition, in 1892, the first clue for the existence of viruses was presented by Dmitri Ivanovsky (1864–1920). His famous experiments with porcelain filters showed retention of bacteria in the residue above the filter but the filtrate was as infectious nevertheless. The name virus (*contagium vivium fluidum*) was coined in 1908 by Martinus Beijerinck (1851–1931).

These new etiological insights involving bacteria, parasites, and viruses, gave an impulse to preventive control of pathogenic MOs in animals, plants, and in or on their derived food products. These insights also fueled further technical development of a methodology to investigate, detect, and screen MOs of which many reviews report, for example [51,52].

### 2.3. Immunoaffinity Principle

#### 2.3.1. Microbial Handles for Analysis

Unique morphological, chemico-structural, biochemical, and genetical characteristics of MOs are opportunities for analytical methods to spot a pathogen sensitively and specifically. The antigenicity of some elements of MOs triggers the immune system of vertebrates in several ways. One of these ways is a humoral response, i.e., the production of antigen-reacting immunoglobulins (IGs) or antibodies (ABs). This reaction is specific between a certain antigen and a particular antibody, and therefore informative. The unraveling of antigenic, but also of biochemical and genetic, differences are the basis for the taxonomy of MOs.

**Figure 3:**
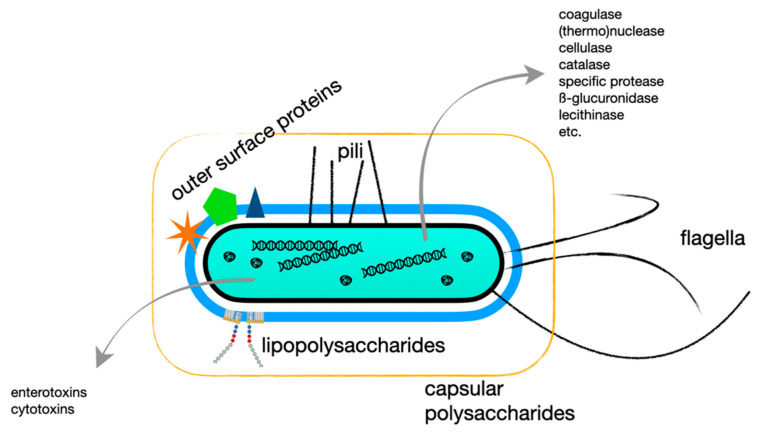
A simplified overview of “handles” of a Gram-negative bacterium analytically available to find a bacterial cell among all other food components. Flagella, H-antigen, are only present on motile cells and when present, it can be a single flagellum or multiple flagella organized mono-/lopho-/amphi-/peritrichously [57]. Lipopolysaccharides, O-antigens, form the outside of the outer membrane of the bacterial cell. Pili or fimbriae, F-antigens, are of little relevance to food microbiologists. Capsular polysaccharides (CPS), K-antigen, can be formed in both Gram-negative and Gram-positive bacteria. A special subtype of the K-antigen is the Vi-antigen in Salmonella. The activity of excreted enzymes is partly specific and used as a marker to identify a bacterium. Analysis of excreted toxins can also be used to trace and identify a pathogenic bacterial cell. The depicted lists of enzymes and toxins are not exhaustive.

In the case of bacteria, ABs generated by a vertebrate are directed against motifs at the outer cell wall (Figure 3), but, in principle, the host can also raise IGs against excreted bacterial moieties. As produced ABs react specifically with certain structures, they can be used for analytical purposes to determine the presence of antigens and thus of a disease-causing agent. Originally, difference was made between heat-labile (proteinaceous) and heat-stable (involving e.g., polysaccharides) antigens. Characterization is now performed using multiple specific antisera against various outer cell envelope structures, predominantly capsular polysaccharides (K-antigens), fimbriae (F-antigen), flagella (H-antigen), and lipopolysaccharides (O-antigens) (Figure 3). *Cave*, the formation of capsular polysaccharides may obscure antigenic cell wall structures for detection as observed for e.g., *Staphylococcus aureus* [53].

Over 150 O-antigens and approximately 50 and 60 K- and H-antigens, respectively, are described for *Escherichia coli*. Of the over 2400 known O: K:H-*E. coli* variants, most are not pathogenic. Identification of *Salmonella* is primarily through its somatic O-antigens (LPS) and has revealed the occurrence of many (sub-)subspecies, so-called serovars. Through shared O-antigens, *Salmonella* serovars are categorized into serogroups indicated by letters. Of interest to food microbiologists are for example zoonotic *Salmonella* serovars belonging to serogroups B, C, and D.

Immunodetermination of *Salmonella* using specifically reacting ABs should be performed carefully as identical O-antigens are also found on other potential food-contaminants such as *Citrobacter freundii* and *Escherichia coli* O157 [54]. For this and other reasons, *Salmonella* subspecies are occasionally further specified by supplementary profiling of the flagellar (H) antigens, which are more specific than O-antigens. Other antigenic *Salmonella* membrane proteins are generally not used for serological analysis as these proteins show cross-reactivity with other Enterobacteriaceae *genera*. In this way, serovars of *Salmonella* are described by a unique combination of O- and H-antigens. This classification is known as the Kauffman-White (-Le Minor) Antigenic Scheme. The list of *Salmonella* serovars is growing with new sub-subspecies verified formally by the WHO Collaborating Centre for Reference and Research on *Salmonella* (WHOCC-Salm). Hitherto, over 2600 *Salmonella* serovars have been described [55] of which less than 100 serovars account for infections in humans. Genomic analyses have shown that the 2600 *Salmonella* strains belong actually to two species, namely *Salmonella bongori* and *Salmonella enterica*.

Some bacteria show relatively fast genetic shifts in their antigenic structures and manifest in many different variants and serotypes. This phenomenon hampers the development of specific binders to facilitate the detection of for example *Campylobacter jejuni* and *Listeria monocytogenes* at the species level [56].

*Campylobacter* spp. does not show a one-to-one serovar-genotype relation, and an isolate can change its serovar over time [57]. This makes categorization and analysis complicated. Nevertheless, the Penner-typing scheme based on antisera against capsular polysaccharides and LPS is used to identify *Campylobacter jejuni* ssp. Jejuni which is the most frequent cause of campylobacteriosis worldwide [57]. It demonstrates that some MOs need specialistic knowledge before one can start to develop, validate or exploit immunological methods.

Detection and identification of parasites rely for a great part on visual inspection (cysts) and microscopic techniques. In general, molecular, i.e., nucleic acid sequence-based (NASB), techniques have poor sensitivity due to a low parasite burden (see also below). Protozoans and helminths are very diverse and do not have generic (morphologic) structures shared between *genera*, family, order, and classes such as bacteria do. Nevertheless, parasites give themselves away by triggering an immune response not only as an intact entity but also by releasing excretory-secretory antigens into e.g., the circulation [58,59,60].

Viruses are actually obligate intracellular parasites. Like protozoans and helminths, viruses are extremely diverse in their structures, genetic compositions and in their ability to infect, persist and initiate disease in a host [61,62]. Any viral protein may provoke the generation of antibodies. However, many viruses have evolved mechanisms to sabotage the arms of the immune system, including those of a humoral response. They do this for example through antigenic drift and shifts resulting in mutation of protein regions that are normally targeted by ABs.

Despite an antigen-antibody reaction is specific, the primary or stereochemical structure of epitopes may be related so that the paratopic loci on antibodies are not able to discriminate different analytes sufficiently, in particular when MOs are phylogenetically closely related. This cross-reaction is a frequently occurring feature of ABs. The developer, producer and end-user deploying an IA method should be conscious of false-positive outcomes caused by this phenomenon.

#### 2.3.2. Antigens and Antibodies as Potential Analytical Tools

Following infection, the immune system can react in various ways, such as through cellular (by releasing e.g., cytokines) and/or by the production of IGs (humoral response) [61,62]. Whatever the response, each can be exploited for diagnostic and other analytical purposes. Cytokines regulate and mediate immunity and are commonly less specific than antibodies to trace infections or to determine the success of vaccination. This review focuses on antibodies reacting specifically with an antigenic structure of a pathogenic MO. The antigen can be a unique biomolecule, structural element, a primary or secondary metabolite, e.g., an excretory/secretory product of the MO (*cf.* Figure 3). An example of a secondary metabolite is a bacterial or fungal toxin. An example of an excretory/secretory product is the ES antigen of the parasitic worm *Trichinella spiralis* used in ELISA serology [63].

Specific bio-recognizing antibodies are produced, isolated, and occasionally purified, and used in a plethora of analytical tests for diagnoses, monitoring, screening, and surveillance. A bio-recognizing antibody used as an analytical tool, cannot be designed by a rational template or code. They have to be produced by immunization techniques. Sufficient specificity and affinity are not guaranteed. Traditionally, antibodies, polyclonal antibodies (PABs), are isolated and concentrated from a mammalian animal exposed to an isolated antigen or an attenuated MO, or is infected under controlled conditions. Factually, these approaches are all vaccinations.

On the other hand, monoclonal antibodies (MABs) are produced ex vivo by hybridoma cell technology developed by Nobel laureates Milstein and Köhler in 1975 [64], which was a huge step forward in the history of immunoassays. Immortalized cell clones yielding ABs with favorable characteristics, such as a high avidity, are picked for MAB production.

MABs are generally rather specific through binding a single epitope only. In contrast, PABs are diverse in terms of e.g., subclasses and have an affinity for a variety of epitopes/antigens, which can improve sensitivity. The tradeoff is that PABs are associated with a much higher risk of a-specific binding by “reading errors” and/or non-specific binding events compared to MABs. The choice of the primary AB, either MAB or PAB, can markedly affect the specificity of the final IA assay. Therefore, MABs and PABs have to be exploited strategically and require different binding conditions in a test set-up.

Alternatively, to take advantage of a multi-epitope binding ability as PABs have, a mixture of MABs binding to multiple epitopes, so-called oligoclonal antibodies (oABs), may improve test performance. In the first report in 1983, this approach was coined “cooperative immunoassay” (CIA) and increased the sensitivity of an assay twice [65]. The exploitation of oABs was re-introduced in several recent studies (see [66] for more information).

Besides ex vivo hybridoma cell technology and in vivo production in mammalians, antibodies can be manufactured alternatively through exploiting phage-display banks [67]. In fact, phage-display technology was used to produce single-chain fragment (scFv) binders from different clones to bind a wide range of *Listeria monocytogenes* serotypes and strains where “traditional” methods failed in many attempts [68]. Finally, specific non-antibody binders, such as aptamers and molecular imprinted polymers (MIPs), as alternatives for antibodies are attracting increasing attention as well.

For analytical purposes, a specific interaction between immunoglobulin and an antigen is exploited in vitro in two ways: either in a direct antigen (Section 2.3.2.1) or an indirect antigen (Section 2.3.2.2), also known as an antibody or serological test. Reference [69] gives the interested reader a brilliant overview of the many different direct and indirect IA assays developed since the end of the 19th century illustrated with authentic pictures and figures.

Finally, the following has to be emphasized here. Any immunoaffinity technique starts with the antigen. After all, no immunogenic structures, no immunological response, no immunoaffinity methods. Consequently, the antigen will determine the quality of the direct and the indirect antigen test.

##### 2.3.2.1. Measuring Principle of Direct Antigen Tests

As described above, the ways to detect the MO of interest are an indirect antigen or a direct antigen test. In a direct antigen immunoassay, isolated specific antibodies bind to probed antigens if present in the sample. The types of samples suitable for direct antigen testing are numerous. A sample can be (a swab of) any part of the (culled) animal, fruit, vegetables, salads, environment (of the farm, abattoir, processing plant, truck, bakery, butcher, supermarket, kitchen), packaging material, but also eggshells, feces, feathers, hair, and saliva. In the last matrix, for example, the authors succeeded to detect zoonotic *Streptococcus suis* serotype 2 through its secreted antigenic extracellular factor (EF) in the broth of cultured swine saliva using a surface plasmon resonance (SPR) biosensor [70].

Selection of a sample type and the moment of sampling should be done carefully, and with consideration of circumstances and implicit factors to increase the chance of a hit. Specialistic epidemiological, etiological and pathological knowledge of a MO, including period and routes of shedding, tissue predilection, is essential for effective microbiological screening. As an example, pathogens are not distributed proportionally over a carcass. It is for this reason that the EU Regulation on microbiological criteria has specified that four sites of a carcass have to be sampled with a minimal surface of 20 cm^2^ in the case of aerobic colony counts or Enterobacteriaceae, or, when swabs are used, at least 100 cm^2^ [3]. In the case of *Salmonella*, an abrasive sponge must sample at least 400 cm^2^ of the most likely contaminated areas.

In direct antigen tests, detection is performed in many different ways not only using the antigenic moieties of the pathogen exclusively. In so-called sandwich assays, the immobilized antigen-binders are MABs but more likely PABs for sensitivity purposes, and a secondary AB, often a MAB for specificity aims, provides a signal (through a conjugated label).

##### 2.3.2.2. Measuring Principle of Indirect Antigen Tests

An infection is recognized by increasing levels of immunoglobulins specifically reacting with the invading MO. The antibodies are of the IgA, IgG, IgM, or IgY (IgG homolog in fowl) class and have different binding characteristics, which are seldomly deployed in food analysis unlike in medical microbiology. Antibodies are found in body fluids, including blood, cerebrospinal (liquor) or synovial fluid and saliva/mucosa, but also in egg (fowl), extracellular fluids, milk and colostrum (lactating animals), and tears. There is a correlation between the concentration of antibodies (titer) and disease burden but this correlation is not strong. Immunoglobulins are (stereo)chemically and biochemically relatively stable, including quite resistant towards proteolytic attack. Therefore, the determination of antibodies can be performed rather long after the onset of death and after sampling.

In an indirect antigen test, responsive antibodies will bind to an isolated (purified) and specific antigen and in this manner indirectly demask the presence of an MO. This testing principle is used predominantly to assess the risks of animals and raw animal products before they are released to the secondary sector (Figure 2) because responsive ABs are a good indicator or biomarker of a (past) infection. The detection of specific antibodies is also named serology, but this designation is avoided here as it can be confused with serological confirmation of an isolated bacterial cell. In many instances, the antigen is (semi-)synthesized or produced in cultured immortalized cells by recombinant DNA protein techniques, such as antigens representing the *porcine* reproduction and respiratory syndrome (PRRS) virus [71].

The diagnostic sensitivity, i.e., the ability to correctly determine infected individuals or populations, of this type of assay is hampered by:

(*i*) Low immunogenic response of the individual animal, and
(*ii*) The so-called seroconversion window.

*Campylobacter jejuni* and *C. coli*, the most frequently isolated foodborne pathogens from humans suffering from gastro-enteritis, colonize the intestinal tract of most animals [72], but these do not show clinical symptoms of the disease. Animals apparently lack specific factors, such as receptors, and/or have an effective immune mechanism [4]. In fact, the lack of an animal model, except non-human primates or genetically or surgically modified animals, hinders significantly scientific research to understand campylobacteriosis [4]. For example, circulating antibodies neutralizing *Campylobacter* cytolethal distending toxin are not developed in chickens, while these antibodies are elicited in humans [73]. So far, no indirect antigen assays are routinely in use for the detection of *Campylobacter* [72].

The course of an infection, i.e., invasion, colonization of a host by a MO and MO clearance, the onset and development of a humoral immune response, and the duration of detectable IGs against the MO are asynchronous and do not match. In accordance, the results of direct antigen tests do not balance well with those of indirect antigen assays [62]. In other words, a seropositive individual may be free from the tested pathogen; a positive indirect antigen test reports in that case a past but cleared exposure to a pathogen. On the other hand, many pathogens, including viruses, bacteria (e.g., *Salmonella*), and parasites (e.g., *Toxoplasma*), can “hide” themselves, for example intracellularly, leaving them undetectable in a direct antigen test but detectable indirectly by specific IGs. Under certain circumstances, such seropositive animals become contagious again and for this reason, they should be traced to prevent contaminated food.

A seroconversion window refers to the time needed for a vertebrate to respond to an infection, *i.c*. the interval after commencement of the infection to produce detectable amounts of immunoglobulins. This window varies considerably between approximately four days and even weeks depending on the bioagent, animal, and the type of matrix in which the IGs are searched. A negative antibody test on an individual is therefore no assurance that this tested individual is free from the pathogen unless the test is repeated (in some cases multiple times) after an appropriate time. But even following an adequate time-interval, the IG-concentration can be less than the limit of detection of the assay. A notorious example is the failing or weak seroconversion of poultry upon infection of *Salmonella enterica* serovars in the C serogroup (O:6), such as zoonotic *Salmonella enterica* Infantis [74], while it is a commonly isolated serovar in laying hens and broilers. This is a major drawback of the technique and one of the reasons why indirect *Salmonella* testing is not popular in poultry monitoring programs.

In addition, IG-classes have different binding-reactivity properties and different anabolism and catabolism characteristics. One must therefore realize that IG-concentration profiles of different IG-classes in different body fluids are similar but not identical. The concentration of *Salmonella*-binding IGs in meat drip or meat juice (see also below), which is an important analytical matrix in national screening programs in several countries, such as Denmark, is for example lower than found in blood serum [75,76]. Although concerns have arisen from the use of meat drip for *Salmonella* monitoring on several occasions [75,76], an important driver to assay meat drip instead of blood serum is that it is practically easier than collecting blood samples at for example slaughter [77,78]. It is also a matrix with high predictive power for the occurrence of HEV in pork [79]. Its use to screen other pathogens, such as the protozoan *Toxoplasma gondii* in other food animals, including goats and sheep [80,81], has been well demonstrated. However, anti-*Toxoplasma gondii* IgG levels in heart, diaphragm, tongue samples vary significantly [82] and test sensitivity using diaphragm juice is 60–77% compared to serum [83]. For the preparation of meat drip samples, these muscle types are nevertheless preferred as they represent a low economic value.

Meat drip is suitable to screen carcasses, but obviously not suitable to screen animals in the pre-harvest phase. Typically, blood samples for indirect antigen screening are collected in this primary phase. However, unlike collecting feces, sampling blood in pigs at a farm gives labor and animal welfare issues. It is therefore that other body fluids, like oral fluids, are investigated as an alternative [84,85]. The use of oral fluids for agglutination tests goes back as early as 1909 [85]. In saliva samples, IgG, IgM but predominantly IgA is found. The antibody concentrations are lower than in serum and methods should be adapted for oral fluids to let sensitivity in line with those suitable for serum or meat drip [84,86]. The choice of the matrix will thus influence the diagnostic sensitivity of the tests. For example, screening of oral fluids collected on cotton ropes hanging in pens for antibodies against the PRRS virus is becoming increasingly accepted. The pigs bite playfully in the rope leaving saliva. A factor to be aware of is that ill animals bite less frequently (thus an indicator by itself) and can be missed by the “rope test”. Although PRRS is a widespread disease affecting swine, not humans, and thus not a food-safety issue, the disease can be spread through careless wasting of food leftovers and using food spills in feed.

For clarity, analytical and diagnostic sensitivity (or specificity for that matter) are fundamentally different terms. Analytical sensitivity refers to the smallest detectable amount of analyte (e.g., antibody in serum), i.e., the detection limit of the assay. It can be assessed by dilutions of commercially available reference samples in samples of negative animals to define the penultimate dilution in which the analyte is no longer detectable or indistinguishable from negative samples. Similarly, analytical specificity is the degree to which the assay does not cross-react with non-targeted analytes in a standard sample. Diagnostic sensitivity and diagnostic specificity determine the probability that a given test result reflects the true infection status of the animal. The values are derived from testing a series of samples from reference animals (which are assessed by the reference method as well).

A way to overcome a disappointing diagnostic sensitivity, even when the analytical sensitivity of the assay is satisfactory, is to screen the seroprevalence of a group instead of assessing the serostatus of an individual animal or product. In this approach, the flock, group, herd, or (products of) carcasses are considered as a single batch within which infection kinetics and dynamics are actually averaged. In other words, assessment of a cluster will normalize results and blot out the impact of false negatives and false positives. As thought fit, this does not imply that samples are pooled before analysis; it means that each individual sample is analyzed separately followed by evaluation of each outcome within the sample series resulting in an overall score for the batch/group.

The chosen sample size is then of principal importance to yield statistically significant results which allow educated decisions on condemning or releasing a batch, i.e., considering it non-compliant or compliant, respectively. Sample size depends on many parameters, circumstances, and decisions, such as prevalence of infection (in a region), sensitivity of the test, (non-)hypergeometric sampling, desired confidence level *et cetera* [87].

Another matter to consider is that, in principle, indirect antigen analyses do not discriminate a seropositive signal coming from a naturally wild-strain-infected animal or a vaccinated animal. It is for this reason that countries having acquired a specific disease-free status, prohibit vaccination against the respective animal disease unless a so-called marker vaccine is used. An example is an immunization against non-zoonotic Aujezkýs disease or pseudorabies virus (PRV) using vaccines containing (attenuated) modified viruses devoid of certain otherwise antigenic proteins [88]. This technique is known as DIVA: differentiating vaccinated from infected animals. Similarly, investigators using NASB methods may miss modified vaccines when they use non-adjusted primers, such as the Riems C-strain of the CSF virus [89].

Finally, some pathogens are typically, not exclusively, introduced *postmortem* in the processing plant or the kitchen and will therefore not provoke an immune response that can be picked up by an indirect antigen test. Examples of such pathogens are *Listeria monocytogenes* or norovirus, but also *Salmonella* by e.g., *postmortem* cross-contamination.

These mismatches between results provided by direct and indirect antigen assays give the impression of false negatives and false positives. This is a needless debate as the purpose of testing, in general, this is risk management, dictates the choice of test approach. The indirect antigen analysis strategy fits well in food quality assurance programs as samples are relatively easily collected and processed with high-throughput and laboratory robotics possibilities. These possibilities facilitate cost-effective monitoring, three months intervals are usual, of pens, herds, and farms providing a risk status.

Specific biomolecules also reveal the presence of MOs indirectly in other ways. An example is the major virulence factor p60 of *Listeria* spp. which it excretes in relatively large quantities. This surface protein biomarker was detected in milk using an electrochemical immunosensor [90]. Among other biomarkers are volatile metabolites which can be profiled at ppb levels using so-called electronic noses (E-noses). *Salmonella enterica* Typhimurium, for example, is recognized by primary and secondary alcohols, and methyl ketones [91]. *Escherichia coli* produces typically large amounts of indoles not produced by other pathogenic or spoilage bacteria [91]. The specific production of indoles by *Escherichia coli* is not surprising, as the bacterium is a prominent intestinal inhabitant and indoles, as degradation products of tryptophan, are causing the characteristic noxious smell of (human) feces. A major drawback of E-noses is that they easily interfere when the composition of the surrounding air changes. Analysis should therefore be performed in rooms with low effluent from other sources. The use of, for example, alcohol to disinfect hands, or a nearby car with a running engine makes E-nose analyses unreliable.

#### 2.3.3. Sample Type and Preparation

##### 2.3.3.1. Sample Preparation for Direct Antigen Tests

Bacterial cells may be stressed, damaged, or otherwise sublethally injured by intrinsic factors such as organic acids, the activity of preservatives, and high salt concentration, or extrinsic factors such as cold or heat. Notwithstanding their bad condition, cells may revive and proliferate in/on a food product under more favorable circumstances, like meat presented improperly at the market lying at elevated temperatures in its protein-rich drip. As tests used for inspection purposes fail to detect the target MO straight in the animal or food product (elaborated on below), bacteria are multiplied first by inoculation of a broth and culturing. This so-called (pre-)enrichment step also offers the possibility to improve the selectivity of the overall method.

There is another reason to execute this culture step. Assuming sufficient sensitivity, a test method could produce false-positive test results, i.e., correct identification but of a non-viable organism which is therefore no longer a public health threat. The culturing step thus not only improves selectivity, but it also prevents wrong conclusions as per definition only viable cells are multiplied.

Overnight incubations in a liquid nutrient at a specific temperature optimal for growth of the target organism(s) and suppression of that of other bacteria are therefore common. In the culturing step, an initial incubation time has to be respected to allow injured cells to resuscitate. It is for this resuscitation interval that bacterial methods are hindered to obtain a fast time-to-result (TTR). In addition, quite some pathogens, such as *Salmonella enterica* group B strains [92], are known to grow slowly. Most food- and water-borne bacterial pathogens require at least 18 h, even up to 72 h, incubation. So-called “same-day” assays, comprising sample preparation, (pre-)enrichment, and detection in a working day (8 h), have therefore to be applied carefully to avoid false-negative outcomes. Notwithstanding, “same-day” methods involving real-time PCR (qPCR) for *Salmonella enterica* determination in carcass swabs and pork, veal, and poultry meat samples show favorable results [93,94].

A means to isolate and concentrate (intact and viable) MOs is immunomagnetic separation (IMS). The enrichment technique involves paramagnetic beads coated with MO-binding immunoglobulins. The microspheres are suspended with the sample, such as incubated broth or food slurry, allowed to capture the target and then collected from the suspension using a magnet. Meanwhile, the particles are washed to remove interfering food particles, (supra)molecules, and any other (competing) MOs. In other words, IMS does not only isolate and concentrate but also purifies the target. The technique is easily combined in automated systems to bring down labor and time and it is a popular step in many rapid methods. In quite some examples, analyte-microspheres complexes are suitable for analysis without further treatment, including traditional culturing, ELISA, molecular biology methods, MALDI-TOF mass spectrometry, and affinity assays. An example is a combination of IMS-beads with a paper-based carrier for color development and colorimetric evaluation using a smartphone. This approach has been demonstrated for *Salmonella enterica* Typhimurium in Starling bird feces and whole milk at 10^5^ CFU/g and 10^3^ CFU/mL, respectively [95]. As an alternative, bacteria can also be isolated and concentrated up to 500 times using anti-*Salmonella* antibodies immobilized on Affi-Prep, which is cross-linked acrylamide support, or on beads in immunoaffinity chromatography [96].

There is little that is perfect *a posteriori* that appeared perfect *a priori*. The IMS technology is bound by the quality of the binder which limits the range of application. It is a challenge to bind e.g., all food-safety relevant *Salmonella* serovars with similar affinities while not binding MO with related antigens. Specificity of the detection method after IMS must compensate for this failure. However, abundant competing non-targeted MOs can occupy the binding sites and prevent the target to attach causing false-negative results in this way. Non-specific binding causes another problem and may cause false-negative as false-positive results as well.

The IMS efficiency is highly dependent on the sample matrix and on the result of the pre-enrichment culture with recoveries, which are disappointing when sampling complexity increases (Author’s experience with *Salmonella* in Pathatrix system; [97,98]). Besides food matrix interference, the costs of IMS beads are an issue in routine food microbiological screening. Therefore, there is a continuous exploration for IMS alternatives for the isolation and concentration of target MOs from large volumes.

As an alternative for classic IMS beads, the magnetic microspheres are coated with (synthesized) binding molecules other than immunoglobulins. For example, bead-linked aptamers have been used to purify *Salmonella enterica* serovar Typhimurium from food samples [99]. Another alternative for IMS, with a long history, is continuous flow separation on metal wires [100].

Whatever steps are needed to prepare a laboratory sample into a test portion, it is without further explanation that treatments have to preserve the phenotypical of genotypical characteristics of the aimed MOs to allow correct determination.

##### 2.3.3.2. Sample Preparation for Indirect Antigen Tests

Indirect antigen tests require samples containing reporting reactive immunoglobulins making the technology not applicable for plant products. Qualified sample types are blood, colostrum, eggs, feces, liquor, meat juice, milk, mucus or saliva, and tears, which can be collected *ante* and/or *postmortem*. Some of the listed matrices contain detectable levels of IgA, such as colostrum, mucus, and feces. However, blood serum and meat drip are used most frequently for indirect antigen (AG) investigations. These materials are superfluously available and relatively easily prepared, handled, and stored.

Whole blood is used seldomly and is treated to yield plasma or, in prevalent cases, serum. Meat drip, also called meat juice, is usually collected from the diaphragm, a muscle with low economic value. It is prepared commonly by placing a piece of muscle tissue (1 to 5 g) in a holder on top of a simple filter in a labeled tube. After freezing the complete set, including holder with tissue, filter, and tube, while, for example, transported to the laboratory, it is thawed just before analysis. Besides cytosol, the drip is composed of extracellular fluid mirroring serum proteins, including immunoglobulins. The matrix is suitable for testing as long as it is stored properly, and denaturing and inactivation of IG by freeze/thaw cycles and heat is prevented (see e.g., [101]). Furthermore, the fat content of the sample should be low as fat and other lipid-like materials can affect the analytical performances of the indirect antigen assay.

A clear advantage of indirect antigen testing is that multiple analytes can be screened in the matrix. Multiplexing bacteria, parasites, viruses, and even residues of pharmaceuticals in a single test is possible [102]. This versatile use and range of operation of indirect antigen tests are difficult to match by NASB techniques. These techniques have a blind spot for MOs giving a low burden, such as parasites and many viruses as well (further elaborated on below). In addition, histochemistry and microscopy in some cases of parasite monitoring (e.g., *Trichinella*) are labor-intensive, time-consuming reference methods for foodborne parasites making indirect antigen assaying a more attractive choice.

### 2.4. Where and How to Measure?

Despite quality and safety control mechanisms, such as HACCP and the good practices of GAP, GHP, GMP, GVP, effective blocking of contamination cannot be realized without analytical tests at critical points in the chain. Sampling at critical points is essential but is not trivial. The food-chain is complex and highly (internationally) organized with extended complexity (Figure 2). Sample type and sampling moment, but also apparent obvious matters like sample handling, quality of sample transport and repository, autolysis, contamination (with detergents, antiseptic materials), temperature fluctuations, UV exposure, influence the (quality of the) outcome of the result. Taking the wrong sample at the wrong moment, and/or label, store and/or transport it inappropriately and the analytical result of the most sensitive, most specific and most accurate microbiological analytical method is worthless (Table 2). Sample keeping and treatment should be optimal for the best result. In addition, analyses are credible when at least:

(*i*) The investigator is adequately educated and trained to perform the analysis.
(*ii*) The place where the test is performed is appropriate and hygienic measures are adequate and not a source for false positive (or false-negative) results.
(*iii*) The test is fit for its intended purpose.

Typically, testing is hindered by:

(1) Asymptomatic carriers of a pathogen that remain unnoticed and are not excluded from the food-chain or not further investigated following a visual sanitary inspection.
(2) The sometimes extremely low microbial dose causing disease in humans which therefore needs very sensitive analytical methods, and
(3) the overwhelming presence of many other, non-harmful, entities obscuring the detection of a disease-causative agent.

These hurdles are relevant to understand to make educated decisions on the samples to be taken at which point in the food-chain, and on the most appropriate analytical method to be applied.

With reference to the first hurdle, *Salmonella enterica* spp. seem to be a ubiquitous part of the chicken’s environment. Among the 2600 known *Salmonella* serovars, only two, *Salmonella enterica* serovars Gallinarum and Pollorum, disease chickens. All other *enterica* serovar infections have only minor, subclinical effects for a short period of time and only when the chickens are young [103], after which they become chronic asymptomatic carriers [104]. The chicken is thus prevalently *Salmonella* tolerant [18]. At the abattoir, an inspector is unable to suspect *ante* or *postmortem Salmonella* in a flock of broilers just by visual inspection unless samples are taken for laboratory investigations. The bird is not or little immune responding to *Salmonella enteritidis* serogroup C either and for this reason indirect antigen testing is not suitable for full *Salmonella* assessment of chickens.

In a similar way, animals infested by parasites may be without symptoms as well. In fact, a parasite giving the least disease burden while producing as much as off-spring is usually very successful in biological terms. In contrast to bacteria, some parasite species can be detected visually by a trained inspector or butcher in particular when they are incapsulated. However, most of these parasites are missed visually as especially viable macroscopic parasites are camouflaged by their meat-like color with little contrast.

Concerning the second critical hindrance, the infectious dose in a meal for healthy persons to get ill is as low as 10 CFU bacteria/g food, 10^3^ bacterial spores, 10 virus particles, or 10 protozoan cysts [105,106,107]. Imminent foodborne pathogens such as *Escherichia coli* O157:H7 or *Campylobacter jejuni* require only an estimated number of 15 cells per gram [50] or 500 bacteria [105], respectively, to disease a person. Compare this with the 1,000,000 *Campylobacter* cells that fit on the tip of a pin. Although it must be said that there is great uncertainty over these numbers.

Legislative norms are for a part based on these low infectious doses. For example, *Salmonella* spp. and *Listeria monocytogenes* should be absent in a 25 g sample of most food products [3]. This sensitivity requirement is a significant and important difference with clinical microbiology. Proving the absence of a single bacterial cell in 25 g material (of a symptomless animal) is of a complete other analytical order and challenge than finding the causative pathogen in an appropriate sample of a diseased patient with clear and informative symptoms. In fact, proving the absence of an analyte is fundamental scientifically impossible. Nevertheless, absence in 25 g sample is a regulatory obligation and comes with problems in the poultry sector for the mentioned pathogens. Scientists worldwide have published a scientific pamphlet criticizing this zero-tolerance [108].

Many reviews and overviews of available techniques for tracing health-threatening MOs in the food-chain see a schism between culture-dependent and culture-independent methods. Such partition is surprising. Suitable detection methods, which can verify the mentioned nadirs must be not only extremely sensitive but at these low levels tremendously selective and specific as well. In routine analyses, they have to deliver this performance on a high-throughput and a low-cost basis without rigorous sample treatment, such as inoculation of a culture broth or intensive sample cleanup in the case of e.g., not cultivable MOs. There is not much indication yet that these so-called culture-independent methods are becoming an industry-standard in the short term.

MOs are usually not dispersed homogeneously in or over a food sample. Hence, the compulsory 25 g sample material increases the likelihood to include pathogens in a laboratory sample. This amount is colossal for modern, advanced (instrumental) methods and needs sample processing to bring it back to the volume of a test portion. IMS is a method used often for this purpose (see Section 2.3.3.1). One of the challenges is that the final test portion has to reflect reliably the contamination status of the originally sampled animal, plant, or product. Occasionally, samples are pooled and a test portion from the pool has then to reflect a whole herd, farm, or batch in a direct antigen assay. It is without further explanation that this way of surveillance or monitoring is very critical for failures. Besides the required sensitivity (SE) and ability to process large sample quantities (10–25 g), this second hindrance reveals another analytical requirement related to the third hurdle as well, namely inclusivity or specificity (SP), i.e., the degree to which the assay does not cross-react with other MOs in the large sample matrix.

The third analytical challenge refers to relatively small amounts of the target analyte amidst large amounts of numerous biomolecules, such as proteins, lipids, and many other (large amounts of) MOs. This means that the analytical method used to trace pathogens should not only be very sensitive but be highly exclusive or selective as well. That is, the degree of the assay to distinguish the target from all other components in the sample should be very high. Usually, this cannot be obtained without sample treatment although modern (instrumental) techniques have introduced tremendous resolving power, such as mass spectrometry and several NASB methods.

### 2.5. Test What, When, for Which Purpose and at What Costs?

A test delivering an apparent simple “yes” or “no” answer to the question “is it safe?”, usually does not provide an answer to questions like: “how safe?” and/or “what is making it not safe?”. More specific questions in the scope of this review are:

(1) Which microbes need to be analyzed and intervened?
(2) Where in the food-chain can these MOs best analyzed and with how many of which (type of) samples? (see also above)
(3) What are the test quality requirements?
(4) What is the most effective test methodology?

With respect to question (1), preferably all hazards are screened simultaneously and continuously. However, a perfect world does not exist and for understandable reasons, choices have to be made. These choices should be primarily risk-based. In a simple approach, risks are assessed as:

*R* = *E* × *P* (+H)

where *R* is risk, *E* is effect, *P* is probability and, optionally, H is the “hype” factor.

This formula exemplifies which MOs need to be intervened upstream in the food-chain. The factors *R*, *E* and *P* are obvious and can be illustrated by *Listeria monocytogenes*: the chance to contract listeriosis is low, but the potential effect of death is substantial (Table 1) and therefore the risk of foodborne *Listeria* is relatively high.

The “hype” factor is an addition to the formula by Prof. Rainer Stephany (Utrecht University, Utrecht, The Netherlands). With this factor, he gave expression to the whether or not justified pressure of media, politics, and/or irrational reactions of laypersons. An example of the H-factor was given here before with the “egg affair”.

Typically, risks are region-dependent. As once pointed out by a South-African delegate to the authors, the supranational screening program applying to products exported from Africa to the EU obligates analyses of typical European risks not being African risks per se, while omitting the screening for typical African hazards. On the other hand, sophisticated legislation providing opportunities for risk-based methodology, risk-based sample sizes, and continuous mutual briefing of scientists and policy makers, can support an effective and efficient surveillance system focusing on all relevant health threats [110,111].

In many cases other than toxigenic *Escherichia coli*, *Listeria*, and *Salmonella* (Table 3), a method cannot deliver simply a yes or no answer. It requires a threshold, not the limit-of-detection (LOD) *per se*, which is preferably scientifically determined. A result below the threshold implies acceptable risks. A result over this level indicates risks that are considered not acceptable. However, such determination of a threshold can also be based on whether the risk can be avoided and how much effort (usually this means “costs”) it takes to reduce the risk. It is for this reason that some thresholds are determined pragmatically as for *Campylobacter*.

The prevalence of *Campylobacter* spp. in broilers is high, especially in organic and free-range flocks [112], and modern production methods are not able (yet) to deliver the degree of biosecurity to prevent disease in consumer at an acceptable level. In addition, the EU allows only limited use of a few decontamination treatments of carcasses which will not completely abolish this microbial risk of poultry [113]. Therefore, elimination of the hazard would mean the prohibition of the production and sale of poultry meat. As this is not acceptable either, so-called food-safety objectives (FSO) were introduced [114,115]. In the case of *Campylobacter* spp. this means that out of a predefined number of samples per batch, a certain number of samples must be less than an established load of the bacterium. In fact, the norm of 1000 colony forming units (CFU) per gram broiler carcass relies on this reasoning (Table 3) [114].

With respect to the second question (2), involving the best position in the food-chain to take samples, how many and which: incidental food contamination must be prevented at all stages of food production, processing, distribution, retailing, and preparation. Although a majority of contamination occurs in the tertiary and quaternary sectors, i.e., mass and home kitchens (see Figure 2), still a significant number of the foodborne infections involve germs originating from a reservoir earlier in the food-chain. These reservoirs can be found in animals used for the production of food and/or in wild animals, including (rodent) pests, living in or near a farm. Occasionally, diseases (*Salmonella*, *Campylobacter*) are transmitted through vectors as flies (*Musca domestica*) [116] and other insects as well. It tells us to screen and tackle the sources of these microbes upstream as early as possible and in the environment close to their source(s).

There is another reason to trace and mitigate risks as early as possible: unpolluted products may be cross-contaminated by a tainted animal, plant, carcass, or food product. Cross-contamination can be prevented when the MO is eliminated or isolated as soon as it is detected. In addition, MOs can multiply themselves in the food-chain when not controlled appropriately. A notable, remarkable and frequently observed phenomenon is that reduction of *Salmonella* prevalence through intervention programs gives opportunity to other *Salmonella enterica* serovars to fill the void created after sanitizing other serovars ([20] and unpublished). Although of another order, but a similar phenomenon nonetheless, decontamination of food products as an intervention step, destroys the colonization resistance of non-pathogenic bacteria and thereby provides an opportunity for new (pathogenic) bacteria (and fungi and yeasts as a matter of fact) to re-colonize the product and introduce new food-safety risks. After all, biological laws and entropy cannot be fooled. Notably, risks, such as that of *Salmonella enterica*, may also come from pet food [9,117]. It demonstrates that the complete human food-chain, including the pet food branch, needs continuous attention, inspection, and control.

With respect to the third question (3), it should be clear for whom food is produced. Analytical quantity and quality requirements for an analytical procedure to examine food safety are stricter when the consumer is a so-called YOPI, *i.c*. belonging to the group of Young (<5 years of age), Old (>64 years of age), Pregnant or Ill (immunodeficient) people. Furthermore, quality requirements also depend on the type of investigation (*cf*. Table 4). Different test requirements are needed for example in a case of (legal) dispute or claim settlement, of a surveillance (aimed, close and short investigation) or of a monitoring (continuous assessment) program.

**Figure 4:**
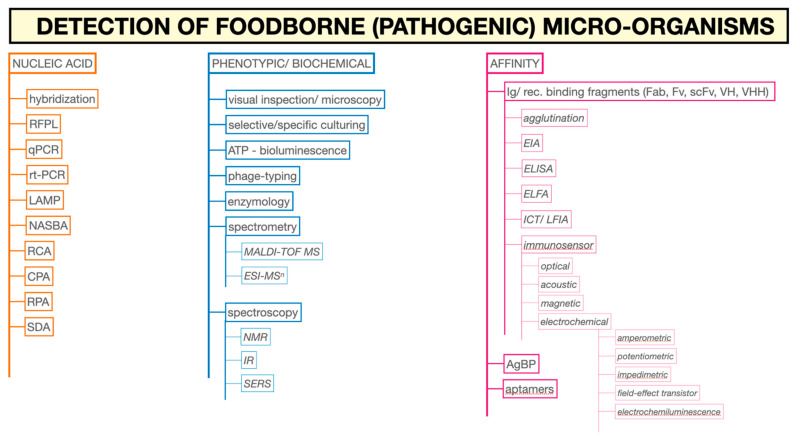
Impression of analytical methods and approaches to find and determine (pathogenic) micro-organisms in the food-chain. It should be noted that electrochemical immunosensors exploit many different detection principles, including amperometry, impedimetry, field-effect transistor (FET), potentiometry. This overview is far from complete. ATP, adenosine triphosphate; AgBP, antigen-binding proteins (not immunoglobulin related); CPA, cross-priming amplification; EIA, enzyme immunoassay; ELFA, enzyme-linked immunofluorescent assay; ELISA, enzyme-linked immunosorbent assay; ESI, electro-spray ionization; Ig, immunoglobulin; IR, infra-red; GC, gas-chromatography; ICT, immunochromatographic test; (RT-)LAMP, (reverse transcription) loop-mediated isothermal amplification; LFIA, lateral flow immunoassays; MALDI-TOF, matrix-assisted laser-desorption ionization time-of-flight; MS, mass-spectrometry; MS ^n^, multi-stage MS; NASBA, nucleic acid sequence-based amplification; NMR, nuclear magnetic resonance; qPCR, quantitative (real-time) polymerase chain reaction; RCA, rolling circle amplification; rec. binding fragments, parts of immunoglobulins obtained by recombinant DNA protein engineering; RPA, recombinase polymerase amplification; rt-PCR, reverse transcription-polymerase chain reaction; SDA, strand displacement amplification; SERS, surface-enhanced Raman-spectroscopy.

With respect to question four (4), “what is the most effective test methodology?”, the spectrum of available analytical strategies is tremendously broad (Figure 4). It is fairly impossible to weigh and compare all these techniques against each other. To assist a user to decide on a method, Table 4 summarizes the characteristics of an ideal analytical procedure. Needless to write that no single existing assay satisfies all listed criteria. The user must weigh a method of choice based on the type of the MO, assessment point in the food-chain, quality of result, throughput, specificity, TTR, etc., not necessarily in this order of importance.

Available analytical approaches can be cataloged roughly and not exhaustively into NASB techniques, immunoglobulin-based methods and biosensor-based technologies, and physicochemical techniques, such as NMR and MS. These approaches are more or less discussed here below.

### 2.6. Immunoaffinity Assays

Immunological methods rely on a specific interaction between immunoglobulin and an antigen to selectively capture, label, and detect antigens associated with target organisms. Besides detection, showing a specific antibody-antigen binding reaction is accepted as a way to confirm the identity of an isolated MO as well. Until the end of the last century, agglutination, EIA, and ELISAs were apparently the only existing IA formats [118,119], but much has changed since then. However, this review does not elaborate on all developments, such as heterogeneous versus homogeneous immunoassays, nor on all possible configurations, such as a sandwich, open sandwich, competitive versus immunometric assays, etc. The interested reader may learn more about these configurations and test settings in [120].

#### 2.6.1. Traditional Methods

##### 2.6.1.1. Direct Antigen Approaches

The first direct antigen methods were developed since the awareness of cellular pathology (see Section 2.2). Agglutination, i.e., macroscopic clumping when agglutinating antibodies in sera react specifically with (bacterial) antigens, was first observed in 1896 by various bacteriologists [121]. This specific antibody-generated clumping of bacteria became known as Gruber–Durham reaction. In the food microbiology laboratory, the agglutination test is hitherto performed by mixing latex spheres coated with target pathogen-binding immunoglobulins with a test cell suspension, which is usually obtained after enrichment. In a positive reaction, conglomerates are visible after some minutes [122]. Typically, tube tests are more sensitive than slide agglutination tests. Since the early bacterial agglutination tests in tubes, on glass slides, and, much later, in microtiter tray wells, the method has evolved into many configurations. For example, gold particles are attached to the agglutinating antibodies to improve sensitivity. In addition, a broader range of applications, including the detection of viruses and parasites, have become available. Besides antibodies, variations were developed using lectins, which bind specific (surface) carbohydrates, and deploying advanced fluorescent emission (see for an example [123]).

Another variation, known as reversed passive latex agglutination (RPLA), is the immobilization of probing immunoglobulins on latex particles which are incubated with food extracts to detect bacterial toxins (the target antigen) to form lattice structures. Examples are the detection of staphylococcal enterotoxins SEA, SEB, SEC, and SED [124] or *Bacillus cereus* enterotoxin [125]. Although RPLA is more sensitive than PCR methods, this method can be less sensitive and less specific than the less easy-to-use cell cytotoxicity tests applied for the same goal [126,127].

In conclusion, immuno-agglutination tests are simple to use, inexpensive, require minimal equipment, and are relatively rapid. One of their disadvantages though is that they consume relatively much antiserum, which can be scarce and costly for some specific (serovars of) MOs.

Lateral flow immunoassays (LFIA) or immunochromatographic tests (ICT), which were sprouting from the continued development of the agglutination test in the 1950s [128], are user-friendly. In ICT tests, IG-binders, including protein A or anti-IG antibodies, are immobilized in a test/capture line on a porous membrane. Other, often gold- or color-dyed particle-conjugated ABs against the antigen of interest are located just under the pad where the sample, such as a portion of (diluted) enriched medium, is put on.

The LFIA tests were one of the first successful attempts with a major impact on fast end-point analysis in, especially, clinical situations. The tests come in different configurations as uncovered immunochromatographic test strips put vertically in a test solution, or “wrapped” in a plastic casing (LFD) which became known to the public as the “pregnancy test”. Examples of the use of lateral flow devices (LFD) is the detection of EHEC O157:H7, *Salmonella*, *Listeria* in different food products [129,130,131,132,133]. However, as explained, an enrichment step by culturing is needed before these tests can be executed reliably. Although the development of the test itself takes typically 10–20 min, a result may therefore still take up to 48 h. A clear advantage is that no (expensive) equipment is needed.

The LFD technique also allows multiplexing in different ways and the interested reader can learn more about these developments in [134]. Multiplexing LFDs can characterize different (closely related) bacteria and viruses and have attracted (veterinary) clinical interest. However, they consume relatively much of a sample, show low reproducibility, and do not offer high sample throughput possibilities.

The development of chromophoric or fluorophores enzyme immunoassays (EIA), enzyme-linked immunosorbent assays (ELISA), enzyme-linked immunofluorescent (ELFA), and chemiluminescence (CLIA) assays in various configurations was a consistent next step in the evolution of microbiological antigen tests. Although the original discovery of ELISA is disputed, the technique saw daylight for the first time at the end of the 1960s in the last century. These assays need more hands-on time than LFIA, although steps can be carried out by robotic liquid handers. Development of ELISA(-like) assays takes longer to obtain results (1–2 h), but they have improved sensitivity, offer quantification possibilities, and allow a much higher sample throughput per time unit.

An improvement of up to five orders of magnitude, detecting as less as 10^−21^ moles (zepto moles; closing into the number of Avogadro!), is obtained by replacing the (enzyme-)label-conjugate by an oligonucleotide that can be amplified exponentially by a usual PCR reaction [135,136]. This technique called immuno-PCR (iPCR) exploits the flexibility and versatility of an ELISA and the signal amplification ability of a PCR reaction. As enzyme inhibiting sample components are washed away in the ELISA part of the procedure, the PCR reaction is unhindered. This combination has demonstrated much better sensitivities for *Clostridium botulinum* neurotoxins A and B in different types of milk than the standard mouse bioassay [137]. The iPCR further evolved into real-time, immunoquantitive variants (iqPCR). One of these iqPCR methods was 10^2^ to 10^3^ times more sensitive than its ELISA counterpart using conventional reporters to detect *Staphylococcus aureus* enterotoxin B (SEB) in different foods, such as coffee creamer, cooked ham, paella [138,139].

The ELFA principle is used, in some cases with better results than traditional methods, to detect *Listeria* spp., *Campylobacter*, *Salmonella*, *Escherichia coli* O157 in various food products [140]. Anno 2020, this measuring principle is available commercially, for example, the VIDAS system (bioMérieux) optionally in combination with IMS to reduce running times significantly.

As most viruses cannot be or otherwise not easily multiplied, their tiny harmful amounts are an arduous analytical challenge to flag products as potentially contaminated. Classic diagnostic methods, including virus isolation, remained principally technically unaltered, or changed and improved only a little. For this reason, but also because viruses are very diverse and change structurally continuously, analytical techniques based upon viral nucleic acid sequences are much more suitable and usually outperform immunoaffinity-based detection approaches. In contrast to traditional tests, NASB methods have advanced dramatically over the last two decades [141].

##### 2.6.1.2. Indirect Antigen Approaches

The first known example of an indirect antigen test is the Widal agglutination test developed by Fernand Widal (1862–1929) using inactivated *Salmonella typhi* as antigen to probe blood serum antibodies in a patient to be able to diagnose typhoid fever [142]. As antigen and serum are minimally prepared, interpretation of a positive result is difficult by the many cross-reactions.

Whatever the indirect antigen assay of choice, the analyst has to be aware of the complex milieu of biological fluids containing components in variable amounts which can affect the assay in a non-specific manner. The matrix may contain soluble receptors and other antigen-binding proteins yielding false-positive outcomes. In other instances, immunoglobulin-ligand interactions may be impaired and may thus produce false-negative results. Moreover, IG-binding proteins, such as complement factors, may occur at unpredictable concentrations and attribute to unreliable results.

For decades, indirect antigen tests were executed prevalently in the well-known 96-wells SBS plates, also known as Microtiter, which is the formal trade name of the plate and spelled like this. Here, SBS refers to the organization of the Society for Biomolecular Sciences which has defined the standards of the plates with 24, 48, 96, and 384 wells. Non-standardized plates with 1536, 3456, and 9600 wells are available as well. The plates were and are used for various indirect antigen ELISA assays, which deployed and deploy still predominantly a chromogenic sandwich configuration. That means, antibodies in a test portion bind to immobilized antigens. In a traditional assay set-up, bound antibodies are then detected by colorimetry after incubation with an enzyme-labeled anti-antibody antibody and chromogenic enzyme substrate. Alternatives for the solid phase of a microtiter plate have brought analytical innovations and some are discussed below.

#### 2.6.2. Advanced Methods

##### 2.6.2.1. Bead-Based Arrays

In 1968, a special sort of microscope was invented in Germany (called “*Impulszytophotometrie*”) to count and differentiate cells. This flow cytometer can detect labeled MOs as in a direct antigen test for food analysis (see for an overview [143]). Whereas the flow cytometer is suitable for particles as large as cells, including bacterial cells, viruses are too small for the usual dimensions of the flow cell of the instrument and cannot be detected directly. The detection of *Escherichia coli* O157:H7 in ground beef is an example of a direct bacterial application [144].

Instead of a static two-dimensional solid phase, such as in SBS Microtiter plates, a solid phase when sufficiently small, can also be coated with a ligand and then suspended enabling fluidics through the whole sample column and other handling with the formed immune-bead complexes. Bead-based assays (BBA) deploy microspheres as solid phase and carrier of a ligand [145]. When mixed with a sample, contact between receptor and analyte is intensified substantially compared to ELISA(-like) assays which largely depend on Brownian motion even when the Microtiter plate is agitated. The fluid is namely almost static at the nanometer scale at which the recognition and binding reactions take place. The improved interaction by suspension of the solid phase provides more binding reactions per volume and per time unit allowing shorter incubation time intervals and correspondingly less non-specific binding events [146,147]. In other words, BBA reaction kinetics are typically fast, reproducible with very good signal-to-noise ratios.

**Figure 5:**
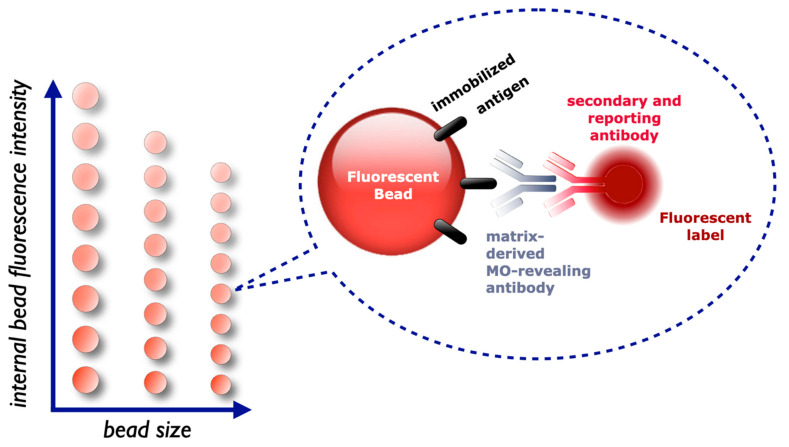
Illustration of an example of a bead or microsphere array used in BBA assays. In the left panel beads are discriminatory in two dimensions: a gradient of an internal color (Y-axis) and diameter of the microsphere (X-axis). Each bead is thus identifiable and can be coated distinctively (right panel). In this case, one of the beads picked from the series in the left panel, is carrying an antigen (black-colored rod shape) to which an immunoglobulin from the analytical matrix (grey-colored Y-shaped structure) is specifically bound. To enable detection of the sample-derived and captured antibody in a flow-cytometer, a secondary, labeled binder is allowed to attach (dark-red-colored Y-shaped structure with reddish orb). The assay is usually developed in Microtiter trays. In the instrument, bead-size, bead fluorescence and label fluorescence of each bead in the suspension are measured and reported. Note that many variations on this test principle are deployed.

The flow cytometer identifies defined beads and measures the signal generated from the binding of an analyte to the ligand immobilized on the microspheres. Actually, the flow cytometer is operated as a flow “beadmeter” performing “beadmetry”. Standardized microspheres come with diverse material compositions available from an increasing number of vendors (see for a list [147]). In most applied configurations, microbeads are dyed with a standardized series of varying intensities of a single or mixtures of chromophores or, preferentially, fluorophores. The flow cytometer can not only identify the color of a microsphere but also discriminate physicochemically closely related microspheres (Figure 5). In other words, besides variation of dyes, other chemical and physical variations, such as quantum-dot tagging, different accurate sizes of microspheres, add dimensions resulting in an array. Correspondingly, the BBA becomes a suspension array assay facilitating multiplexing. Systems with up to 500 distinguishable bead-ligand-analyte combinations (multiplexing) are possible. An advantage of BBA over many other multiplex techniques: a bead-ligand combination, a so-called “set”, is easily replaced, added, or removed from an array of beads.

The multiplexing feature has made BBA assays *en vogue* in many life-science disciplines, including food safety and veterinary diagnostics [148,149,150]. The technique is for example suitable to distinguish naturally infected animals from those immunized with modified vaccines in the DIVA approach, such as for foot-and-mouth disease and Rift Valley Fever virus [150].

Something to notice, however, the resolving power of the instrument may be insufficient to distinguish the spectral character of closely related beads, either by the interference of sample contaminants, poor calibration, and critical performance of the instrument. In addition, low-quality beads may have overlapping characteristics not distinguishable by a properly functioning instrument. One of these failures may result in “blurring” and “tunneling” of signals from the correct channel to the next measuring channel of another bead. Consequently, an analyte is identified and quantified incorrectly as another analyte (authors’ experience).

Multiplexing offers a possibility to simultaneously detect: (i) complete, intact MOs with respect to multiple virulence factors, such as for serotyping, and/or (ii) multiple, different MOs or their freed moieties/excreted elements in a single sample in one analysis run. An example is the simultaneous analysis of staphylococcal enterotoxins SEA, SEB, and SHE in (cottage) cheese, meat broth, minced meat, milk, and omelet [151]. The performance of this BBA method was comparable to a frequently used commercial ELISA and the test was able to declare the products compliant or non-compliant with Commission Regulation 2073/2005 [3,151]. An overview of direct antigen BBA (multiplex) assays to screen a range of animal diseases and clinical biochemical parameters in various animals, including horses, rodents, ruminants, swine, and mycotoxins in feed is provided in [150].

Microspheres suitable for BBA assays come also as paramagnetic variants. The synergy with IMS increases sensitivity and specificity. An example of this approach is a direct antigen multiplex assay for the determination of pathogens and disease biomarkers of *Bacillus anthracis*, *Francisella tularensis*, *Yesinia pestis* causing anthrax, tularemia, and plague, respectively [152]. Another example is the same-day (≤7 h) detection of *Escherichia coli* O157:H7 in spinach at a detection limit of 0.1 CFU/g using magnetic microspheres [153].

Not only beads are modified but the flow cytometer is altered as well. It may contain a magnetic field to immobilize magnetic microspheres in a layer for optical stability so that a CCD camera instead of lasers can be used and more beads at once can be analyzed [145,148]. An example of a direct antigen multiplexing assay using a CCD camera is the typing and classification of O26, O55, O78, O118, O124, O127, O128, O142, O145, and O157 antigens on pathogenic *Escherichia coli* [154]. *Mutatis mutandis*, bead arrays are also used in a planar format in so-called “-omics” technologies enabling thousands of tests simultaneously while each of these tests analyzes a range of molecules in parallel [149].

Indirect, instead of direct, antigen suspension-array assays have shown their merits for screening anti-*Salmonella* antibodies in *porcine* sera [155,156]. A BBA assay for anti-*Salmonella* antibodies in egg yolk to determine the *Salmonella* contamination status of a layer flock showed a good relationship with the infection status of the probed flock [157]. Here, the collection of eggs makes personnel taking blood samples redundant and contributes to better animal welfare. In fact, the collection of eggs for surveillance programs can be executed at egg-packing stations [157].

The legislative and mandatory method for finding the parasitic worm *Trichinella spiralis* is artificial digestion and microscopic examination of pooled pork samples [158]. The method is antiquated and in proficiency tests, the false-negative rate can vary between 11% and 100%, in particular when the parasitic load is low and when the parasites are in their pre-stadium of encapsulation [159,160]. In an animal experiment, an indirect antigen BBA showed 68% diagnostic sensitivity and 100% diagnostic specificity [161]. Despite the better performance, acceptance of indirect antigen assays is complicated because of the seroconversion window, discussed here above (Section 2.3.2.2), which may indeed declare meat from an individual animal falsely negative. Nevertheless, anti-*Trichinella spiralis* antibody screening is a high-quality aid for monitoring exposure to the worm and epidemiological studies [63].

Interestingly, the *Trichinella* BBA method was combined with finding antibodies against *Toxoplasma gondii* in a multiplex assay. This combination is not without reason. The *Toxoplasma gondii* parasite is an indicator of the level of hygiene in the pre-harvesting phase, as it reflects the contact of pigs with their extramural environment [159,160]. The parasites also share routes of infections, although *Toxoplasma gondii* more omnipotent than *Trichinella*, which makes *Toxoplasma* screening only more favorable. Moreover, *Toxoplasma gondii* is ranked as zoonotic MO with one of the highest disease burdens in The Netherlands [162] and USA [163], while it is very persistent and prevalent in the environment as well. Screening *Toxoplasma gondii* may not only help to intervene *Trichinella spiralis* but *Toxoplasma gondii* itself as well.

So far, many multiplex BBAs for pathogen analysis are not IA-based but are built on a combination of microspheres and analysis of bacterial genetic material [145]. A commercially available NASB suspension array multiplex kit for human stool detects *Campylobacter* sp., *Clostridium difficile* (toxin A/B), *Escherichia coli* O157, enterotoxigenic *Escherichia coli* (ETEC) LT/ST, Shiga-like toxin-producing *Escherichia coli* (STEC) stx1/stx2, *Salmonella* sp., *Shigella* sp., *Vibrio cholerae*, *Yersinia enterocolitica*, human adenovirus serotypes 40 and 41, norovirus GI and GII, rotavirus A, *Giardia*, *Cryptosporidium* and *Entamoeba histolytica* [145].

##### 2.6.2.2. Immunosensors

**Figure 6:**
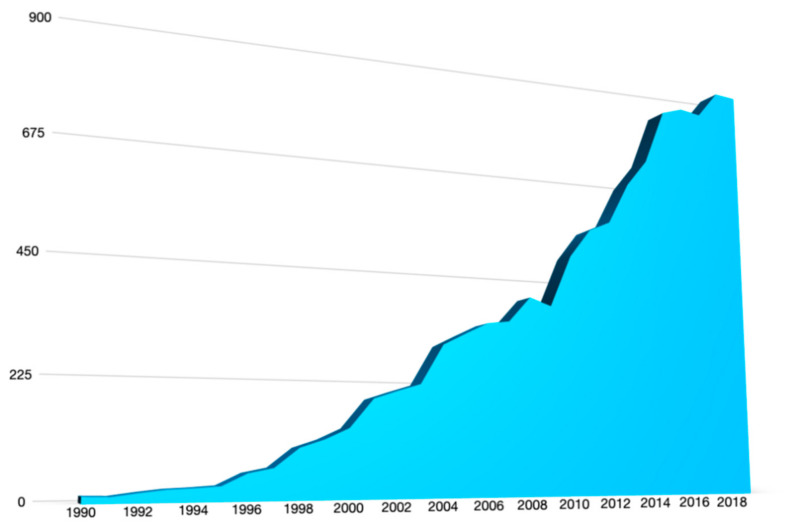
Number of publications per annum returned by PubMed when using search terms “biosensor” and “bacteria” [181].

Biosensors exist since 1962 [164] and hitherto include immunosensors, genosensors, aptasensors, phagosensors, etc. Here, the type of sensor is referring to the ligand attached to the sensor surface, namely immunoglobulins, oligo- or polynucleotides, aptamers, and bacteriophages, respectively. The word “sensor”, i.e., *sentire* (Latin), is referring to something that “perceives” changes in its environment. The instruments are coming into vogue and have evolved at a tremendous pace. In line with this evolution, instruments biosensing (pathogenic) bacteria have attracted increasing attention reflected by almost 11,000 scientific publications since 1990 (12 publications in that year) of which half in the last six years (Figure 6). Roughly a quarter of the publications deal with the analysis of bacteria directly and indirectly in food or animals producing food. It is fairly impossible to give a comprehensive overview of all developments and applications. This paper does not desire to discuss limit-of-detections (using mostly spiked samples), chemical, microbiological and technical details, it aims to place them in the context of current and future practice of producing safe food. If the reader is interested in such details, he/she is kindly referred to reviews attempting to give such exclusive overviews as in recent [165,166,167,168,169,170,171,172,173,174,175,176,177,178,179,180].

There are multiple rationalizations for the spectacular growth of biosensor methods in food microbiology. Biosensors promise fast, ambulant, real-time measurements by a portable device. Many of the systems can split a flow over multiple channels on a single sensor or can combine multiple sensor surfaces in a single flow giving the means to combine the analysis of more than one analyte, indicator, parts, or whole cells. Multi-analyte testing not only involves the determination of e.g., *Salmonella* and *Listeria* in a single analysis run but the determination of species, subspecies, subsubspecies, and even strain as well. Such analytical aims might be accomplished in combination with predictive rationalized mixtures of multiple individual antisera with known binding profiles [66].

Biosensors contain an interface, the sensor, which includes a transducer generating an electronic, quantifiable, and recordable signal. An electronic signal is generated directly or indirectly when binding reactions at the sensor surface change its acoustic, electrochemical, optical, piezoelectric, or thermometric behavior. The reaction is that of the analyte with biologically derived or biomimetic material, such as enzymes, antibodies, cell receptors, lectins, and other (engineered) polypeptides, aptamers, (lipo)polysaccharides, or poly/oligonucleotides. The binders are immobilized on the sensing surface causing the following reaction [182]:

L + A ←→ LA + B_label_ ←→ LAB_label_ + effectuator → SENSOR DETECTABLE EFFECT

Is the reaction in biosensors relying on the use of labels, where the effectuator is a substrate, photon or (conductive) particle etc. providing a measurable response in combination with the label.

or:

L + A ←→ LA → SENSOR DETECTABLE EFFECT

in the case of label-free biosensing.

In these formulae L is a ligand, A is analyte and B_label_ is the labeled binder, such as a labeled anti-analyte antibody.

Many of the machines produce a real-time signal, thus an observable signal while the reaction at the sensor surface is taking place. In fact, this distinguishes many immunosensors from most other IA techniques which are black-box, end-point measurements. Quite some immunosensors function without labeling a molecule to obtain a signal, which is common in all non-biosensor IA methods. Label-free measurements are preferred, as (non-reproducible) labeling chemistry potentially degrading biomolecules can be skipped. Furthermore, labels are notorious for steric hindrance and attachments to active sites destroying the function of the modified molecule. Surface plasmon resonance (SPR) biosensors are an example of such label-free systems and offer the possibility of multiplexing by two-dimensional arrays or multiple channels flowing and probing simultaneously [183]. Besides label-free, SPR measurements are real-time, and a skilled operator predicts the type of binding, for example, binding of IgG or IgM to an immobilized antigen, by judging the binding dynamics while the reaction occurs and the sensorgram is recorded (authors’ observation). Another advantage of real-time measurements is that a run can be stopped untimely if no reaction occurs. This increases sample throughput in particular when the negative sample rate is high also because regeneration of the surface can be omitted.

When aiming for direct immunosensor assays, it should be realized that the size of a rod-shaped bacterial cell is typically 1 µm × 3–5 µm. In some devices, the channels are not much wider than these measures and bacteria can clog up the fluidic parts. In addition, the bacterial size can limit mass transport in a flow, i.e., diffusion of the whole bacteria to the sensor interface, giving a lower response than expected. To overcome this limitation, some methods use boiled bacteria to degrade the cells and enhance sensor response [184,185].

A bulky cell or a large cell part captured to sensing surface hinders sterically the capturing of another cell (part) [186]. In addition, when captured, shear forces generated by the laminar flow in the instrument can be stronger than the energy of the antigen-antibody bond further reducing sensitivity by loss of complexes [96,186]. Other considerations when developing and using direct antigen immunosensor assays are the length of the linker molecule that attaches the anti-target antibody to the sensor surface. Variation of the binding chemistry has a significant influence on selectivity and sensitivity for bacterial cells [166].

Unfortunately, in SPR biosensors, the size and mass of a captured bacteria do not contribute completely to a final response. SPR sensing takes namely place within approximately 700 nm distance from the sensor surface while bacteria are much larger [185,186]. The sensing electromagnetic field decays exponentially until this distance. In addition, immobilization chemistries cut another 10 to 100 nm of a specific signal near the surface layer, i.e., the most sensitive part. A solution for these signal limitations has been developed through so-called (grating-coupled) long-range surface plasmons enhancing the sensitivity for *Escherichia coli* 4 orders of magnitude [187]. Despite all challenges, 53 *Salmonella enterica* serovars were detected in avian feces and breast meat at 22 CFU/g or 1.7 10^3^ CFU per test portion, while 30 non-*Salmonella* species did not give interference [184]. Other examples of successful direct bacterial SPR detection are that of *Campylobacter jejuni* sp. Jejuni, *Escherichia coli* O157:H7, *Listeria monocytogenes*, *Staphylococcus aureus*, *Vibrio cholerae* O1, and *Yersinia enterocolitica* [185].

An indirect antigen SPR immunosensor method was developed to verify the *Salmonella* vaccination status of chickens [186,188]. Using this method involving LPS (O) and flagellar (H) antigen ligands, a linear response in chicken serum was acquired for antibodies against *Salmonella enteritidis* and *S. typhimurium*. Immunoglobulins against *Salmonella enterica* serovar Enteritidis were gauged in egg yolk using an SPR immunosensor to assess the infection status of non-vaccinated layer hens [189]. The method showed favorable sensitivity and specificity performance compared to ELISA methods. Similarly, an indirect SPR antigen assay based on immobilized LPS isolated from *Salmonella enterica* Typhimurium was used to probe *porcine* sera [190].

Like SPR, ellipsometric, surface-enhanced Raman scattering (SERS) and many other optical biosensors [175], impedimetric biosensors can be used label-free. Impedimetric biosensors respond to changes in effective resistance as a result of interfacial events. Sensitivities are as low as 10 CFU/mL of *Bacillus cereus*, *E. coli* O157 and K12, *Listeria monocytogenes*, *Salmonella*, *Shigella dysenteriae*, etc. in various food products, including chicken and milk (see for an overview [173]).

Amperometric and voltametric biosensors (Figure 4) are operated with non-labeled or labeled molecules using different voltametric regimes and reporting limits of detections of 10 CFU/mL and higher for various pathogenic bacteria in different food products, including dairy, eggs, fish, poultry and, remarkably, licorice [173,191]. An overview of developments and applications of electrochemical immunosensors to detect directly *Salmonella enterica* can be found in [192].

Most electrochemical biosensors rely on a label that is often horseradish peroxidase (HRP) [176]. The enzyme catalyzes the decomposition of various substrates while producing detectable electrons. Although some of these systems meet impressive analytical sensitivities, they have relatively long development times and/or are have a limited range of applicability. In an intriguing voltametric biosensor application, the ligand, *viz*. immobilized, specific peptides, is the recognition site and substrate for *Staphylococcus aureus*- and *Listeria monocytogenes*-specific proteases which cause an electrochemical response upon proteolytic cleavage of the ligand [193].

An attractive development in the sensor field is the accessibility of printing technologies enabling (in house) reproduction of electronics at low cost [194,195,196]. Furthermore, the availability, usability of, and possibility to integrate (magnetic) nanomaterials, carbon nanotubes, or nano rods in biosensors in, in particular, electrochemical biosensors is noteworthy [168,171,197]. Nanomaterials offer large surface-to-volume ratios and thereby enhanced interactions and reactions and thus selectivity, sensitivity, and speed. Not only interaction is improved, but nanomaterials also progress the function of the transducer in some sensor types [166]. Examples are the detection of *Escherichia coli*, *Listeria* spp., *Salmonella* spp., *Streptococcus* spp. and *Vibrio parahaemolyticus* in various food matrices [166,172].

Of the non-immunoglobulin binders in combination with biosensors, aptamers and molecular imprinted polymers (MIPs) are attracting the most attention to capture pathogens, including bacteria and viruses [198,199,200,201,202]. The alternative binders are relatively expensive and it can take effort and time to develop and produce sufficient amounts of the pure binder. The xenobiotic molecules are generally more stable than antibodies towards oxidation and heat. Although not degraded by proteases, aptamers are susceptible to nucleases. A potential beneficial characteristic in electrochemical biosensors is the combined negative charge formed by the phosphate constituents in the oligonucleotide backbone of aptamers [166].

Very interesting and exciting is that quite some studies explored the mounting of a smartphone onto biosensors. In this way, the mobile phone is a power source, detection facilitator (LED flashlight), detector (camera), signal converter (computer), and signal recorder (compute and memory storage) part in a sophisticated, portable biosensor system [203,204,205].

##### 2.6.2.3. Microfluidic Devices

Further evolved immunosensors arose from combing and integrating different mechanical and biological techniques for sample preparation, processing, and analysis in a single miniaturized liquid flowing device which is generally known as lab-on-a-chip (LoaC). Solvent application is through micropumps or capillary force and uses hydrophobic stops. In these microfluidic devices, the analyte is immune-concentrated and detected using measuring principles as described above for biosensors (Section 2.6.2.2). In general, these compact platforms offer improved limit-of-detection, reduce human errors, enhance the accuracy of measurements, and are less affected by food matrix components [206]. However, the practice is wilful due to complex food matrices and the small sample volume to meet required selectivity and diagnostic sensitivity. A solution to increase surface area is the integration of anti-pathogen ABs-conjugated (magnetic) nanoparticles, which were used in combination with a LoaC to detect *Escherichia coli* and *Salmonella typhimurium* [207].

Concurrently, attempts are made to give these small systems also multiplexing abilities, which is a challenge by the requirement of the growing number of channels and valves with the number of analytes in usually two-dimensional systems [208]. Indirect antigen assay cassettes have been engineered for detection for anti-HIV and anti-*Treponema pallidum* (cause of syphilis) antibodies in 1 µL whole blood giving results in 20 min. The “mChip” was tested on hundreds of humans in situ in the field in Rwanda and performed equally well as the reference tests in a laboratory [209]. The assay is not suitable for high throughput.

Alternatively, lab-on-a-disc (LoaD) uses centrifugal forces to “pump” solvents and sample over the device. Although most LoaD applications involve LAMP for pathogen detection, IA assays in combination with SPR detection and smartphones have been described as well [210]. For screening purposes, LoaDs appear an interesting alternative for LoaC, as it offers high-throughput possibilities with more flexibility in assay design and multiplexing [208]. In the context of this review, there is a LoaD feature superior to for example LoaC systems: LoaD is virtually indifferent to sample properties like viscosity and surface tension. In the authors’ hands, these properties, which tend to vary considerably per biosample, were often an issue, even after substantial dilution, in developing and validating advanced IA assays.

Generally, the microfluidic device technology is not matured for full routine applications (yet). There are a few unresolved issues which include problems with reproducibility, specificity, and stability and which have to be tackled before microfluidic systems can be considered to have passed their infancy.

#### 2.6.3. Concluding Remarks on IA Assays

Whatever the binder and whatever the test configuration, in all cases the ligand should be immobilized correctly and reproducible to the interface, which can be a sensor surface or solid phase of carriers as in for example BBAs, ELISAs, LFDs. This means, that the ligand is preferably attached covalently or near covalency as in a streptavidin-biotin linkage, and its antigenic determinant or binding complementary regions are orientated towards the liquid in which the analyte is present. The attached ligand should not be inactivated by the immobilization reaction and the ligand should not autoconglomerate/denature on the sensor surface.

Another issue to note is the label used to acquire a detectable and recordable signal. Enzymic conversion of a substrate into a detectable signal follows Michaelis–Menten kinetics. The range of linearity and the window between the minimum and maximum signal is limited. Action limits, the limit of detection, and thresholds levels are often based on optical density in standardized ELISA protocols. Extended ranges are offered by other labels such as fluorophores.

Furthermore, AB-AG binding reaction dynamics are different in ELISA, BBA, LoaC, or immunosensor. Consequently, results are usually not intercomparable without intensive mathematical data massage. To obtain congruency of method outcomes, a significant number of results generated by the alternative and by the reference method have to be regressed to acquire a reliable formula for superimposition. This is a challenging and complex task, as both reference and alternative methods show, besides different reaction and signal dynamics, variance of results. This issue is also addressed by certification and accreditation bodies that request to meet their norms based on traditional, reference methods.

Concerning the difficulty of interpretation of results, it is remarkable how many failures are reported for the apparent fail-safe and easy-to-use LFD. Subjective visual interpretation of the test lines, in particular when they are vague, causes quite some ambiguities [211]. Vagueness can be a result of a contaminated sample, such as blood in nasal fluids in the case of SARS-CoV-2 LFDs. Other failures include inaccurate use of a dropper and thus varying amounts of applied sample. Users applying several loads of sample to the same LFD device deteriorating the carrier are also observed. These issues are not to discourage, but to make the reader aware of the unexpected when developing, evaluating, or executing an assay.

### 2.7. Available Test Principles Other Than Immunoaffinity

Despite the focus on immunoaffinity techniques, a few alternative test principles are discussed shortly as they are closely related or because they can be combined (also referred to as ‘hyphenated’) with IA techniques.

#### 2.7.1. Bacteriophages

Bacteriophages have several intrinsic and desirable characteristics which make them very suitable for the detection and identification of bacteria [212,213]. Their natural specificity and ease of production and use make them very attractive for food bacteriology and promise some solutions for current analytical challenges. In particular, endolysins, i.e., peptidoglycan hydrolases recognizing specifically the bacterial peptidoglycan layer, are of much interest for the design of innovative affinity assays detecting bacteria [214]. Nevertheless, although much progress has been made, only a few bacteriophage-based methods have turned into (clinical) diagnostic devices so far [213]. A major drawback is namely the genetic drift and shift of the target bacteria and of the bacteriophage itself, which, if one thinks about it, is logical or otherwise the bacteria and/or the bacteriophage had not survived and existed anymore. Furthermore, most bacteriophages are very specific and not suitable to screen the huge diversity in bacterial *genera*, species, subspecies, and strains.

#### 2.7.2. Nucleic Acid

Genetic (isothermal) amplification and detection technologies have developed at an impressive pace in food microbiology [215]. They comprise second, also called “next”, and third-generation sequencing technologies. In general, NASB techniques will outperform the analytical sensitivity and specificity characteristics of IA assays. Speciation for example is better by PCR than by IA. When tracing viral pathogens in the food-chain in the case that (serum) antibodies are not available anymore for an indirect antigen test, NASB approaches are factually only suitable.

Despite better analytical sensitivity, the diagnostic sensitivity of PCR can be unsatisfactory. A single tube nested-PCR, of which positive results were confirmed by qPCR, found only 18 *Toxoplasma gondii*-positive goats and sheep, while the indirect antigen ELISA found 79 of the 278 sampled animals [81]. Sensitivity of *Toxoplasma gondii* PCR was criticized earlier [83,216,217]. In these studies, indirect antigen ELISA of serum from naturally and experimentally infected swine clearly outperformed direct, semi-nested PCR and qPCR methods [83]. While rigorous sample cleanup increases PCR sensitivity [218], the procedure is unattainable for testing on large scale [159].

An important disadvantage of NASB assays which can hinder inspection protocols is that they generally do not make a difference between viable and non-viable MOs, in most cases bacteria and parasites, without prior appropriate sample treatment. Degradation of two genes of defunct *Yersinia enterocolitica* comparable to 1 log unit reduction took between 0.5 h in chicken meat and 120.5 h on rinsed pork [219]. In other words, a NASB test without proper sample cleanup or treatment does not discriminate well which products represent a real health threat upon a positive signal.

Decontamination of fresh meat will leave traces of the nucleic acid signature of the killed pathogen(s) possibly recognized unintentionally by a NASB method. In addition, NASB tests, and actually other alternative measuring principles as well, are not suitable to test products against legislative norms expressed in CFUs. The norm for *Campylobacter* in broilers meat, 1000 CFU/g (Table 3), cannot be verified with a NASB method, although droplet digital PCR (ddPCR) seems to offer a solution for this problem (see next paragraph).

Another problem is the inhibiting sensitivity of the enzymes towards components, such as bile salts, EDTA, acriflavine, MgCl_2_, high protein, and fat concentrations, in complex analytical matrices, like feces or food. A solution for this problem comes from (droplet) digital PCR techniques producing and analyzing ideally a single bacterial cell per droplet [220]. The platform is likely able to quantify foodborne bacterial pathogens without sample treatment [221], although it has not been demonstrated how the approach can test the obligatory 10–25 g sample for the presence of unwanted pathogens. In addition, the technology has not convincingly demonstrated yet whether it can discriminate viable from non-viable pathogens either.

The high specificity of molecular methods can be a disadvantage as well. Recently, the analytical risk of a high accuracy became apparent by the SARS-CoV-2 UK variant discovered beginning of December 2020. This variant was poorly picked up by the S-gene target RT-qPCR assay, which was used commonly in routine laboratories [222]. The probe used in this assay attached weakly to the new variant target.

#### 2.7.3. Physicochemical Approaches

Physicochemical instrumental techniques to analyze MOs have been used for a long time, but not routinely. For example, gas chromatography can profile fatty acids of foodborne bacteria [223]. This instrument combined with mass spectrometry (GC-MS) can trace sensitively and specifically microbial markers in/from food, including metabolites and volatile organic compounds (VOCs) [224,225].

In clinical microbiology and increasingly in food microbiology, matrix-assisted laser-desorption ionization-time of flight (MALDI-TOF) mass spectrometry (MS) is becoming a routine methodology to detect and identify pathogens (and spoilage MOs). Alternatively, electrospray ionization (ESI) MS has also found its way into microbiological investigations [226]. These techniques bring characteristic (fragments of) biomolecules of the MO into the gas phase after they are ionized. Separation of the ions on basis of their charge and molecular mass delivers a unique mass spectrum revealing the molecular makeover of an MO in qualitative and quantitative terms. The resulting mass spectra of microbiological samples are very complex and reference databases, data massage, and evaluation software for a workable interpretation are imperative. Nevertheless, these techniques are increasingly able to distinguish morphologically and even genetically similar (sub)species.

Besides spectrometry, variations of Raman and Fourier-transform infra-red spectroscopies, sometimes also hyphenated with other techniques discussed here, are recurrent technologies which fingerprint whole bacterial cells [52]. For example, microfluidics-integrated SERS using gold-surfaced nanoparticles coated with MABs against *Listeria monocytogenes* also capturing *L. innocua*, detected the pathogen in less than 2 min at 10^4^–10^6^ CFU/mL [227].

In general, reported advanced (hyphenated-)spectroscopic techniques are apparently still in an experimental phase. They need purified pathogens at relatively high concentrations. Field applications, i.e., running routine analyses in an agricultural, veterinary, or food safety laboratory, and diagnostic validations of any spectroscopic technique in food are difficult to find. Despite what the developers often claim and promise, namely fast, powerful and reliable alternatives for traditional procedures, spectroscopic methods need lengthy (pre-) enrichment steps and have not shown convincing diagnostic sensitivities or specificities (yet).

## 3. Reliability of Results

Analytical methods blooming from the “food microbiology” field in the 20th century were formally and internationally classified in ISO 07.100.30. This list slowly extended with, at the moment of their inclusion, alternative methods. Anno 2021, this list contains 119 protocols for validation, classic culturing, EIA, HPLC (of histamine as a result of bacterial activity), and qPCR methods for various foodborne pathogens.

It was not without effort to convince the authorities to acknowledge quality assurance and validation protocols for innovative alternative methods. Nowadays, various programs for validation of alternative methods, including LFDs, BBAs, immunosensors, involving detection or determination of MOs in the food-chain have been initiated worldwide. Most of these programs are based on ISO 16140. Examples are programs offered by AFNOR (Paris, France), MicroVal (Delft, The Netherlands), NordVal International (Nordnes, Norway), AOAC-RI (Rockville, MD, USA), and UKAS (Staines-upon-Thames, UK). These and other national or regional accreditation bodies, such as the American Association of Veterinary Laboratory Diagnosticians [228], Collaborating Veterinary Laboratories [229], and European Association of Veterinary Laboratory Diagnosticians [230], will help maintain the validity of diagnostic results from e.g., (novel) IA procedures.

### 3.1. Method Validation

Before any validation or verification process starts, the user should answer the question of whether the test will deliver the requested results in the given circumstances, such as geographical region [231]. The latter refers to a situation not as in Gertrude Stein’s 1913 poem “Sacred Emily”: “A rose is a rose is a rose”, but *bovine* samples from Northern European or from Southern African cattle will give other test results [231]. A similar phenomenon was observed for samples from Dutch and North-German pigs in a BBA assay (dr. Bergwerff, unpublished).

Evaluation of an assay to determine its fitness (performance characteristics) for particular use in the diagnosis, monitoring, surveillance or trade, is essential to ascertain the integrity and reliability of test results. After all, public health and socio-economic impact are often too high to allow avoidable false results. Furthermore, test results should be transparent, understandable, acceptable, and legitimate for any third party, including for a court of law when results or safety of products are disputed. In the case assays are used to test compliance with legislative norms, a validation study is compulsory and not non-binding. Many governmental and non-governmental, national, and international organizations and institutions have devised obligatory validation and verification programs according to recognized international quality standards (ISO). These programs should maintain administrative, management, and technical performances for various diagnostic tests at a high level of accuracy and uniformity. An excellent overview of method validation of assays for the determination of immune status after vaccination and of infections in animals is given in [231]. A lucid description of issues involving calibration and standardization of immunoassays is found in [232].

The variables that affect an assay’s performance can be grouped into three categories:

- Sample: Host-MO interactions affecting the composition of the sample and its analyte concentration. Surprisingly, this can thus be different from one geographical region to another.
- Assay system: Physical, chemical, biological, managerial, acclimation, housing, and technician-related factors affecting the capacity of the assay to detect a specific analyte. Here, sources of errors are not necessarily random and independent. They are for example laboratory effects, method bias, matrix variation effects, random and systemic errors of measurement, run effects, or bias.
- Other sources of errors that affect the capacity of a test result to predict accurately the contamination status of animals, food products, plants, or populations relative to the analyte in question.

With bioagents, such as MOs, the “true” content in complex biomatrices, such as feces, serum, or food, can never be determined exactly. Outcomes from an alternative method are therefore commonly compared with those of the gold standard or reference method. To pass validation, this comparison has to demonstrate equal performance in terms of relative specificity (SP), relative sensitivity (SE), and relative accuracy (AC). Here, lies a hidden potential disappointment. Alternative methods with e.g., increased sensitivity or increased specificity compared to their respective reference methods produce more “false positives” or “false negatives”, respectively.

Commonly, a reference method is recognized, respected, and results undisputed. The reference method is the outcome of a culmination of reaching a consensus in the scientific field of the best available assay at the time of evaluation under given, reasonable circumstances. This is a long and complex process to converge opinions and to reach a common agreement of understanding on a reference standard between the food industry, (veterinary) public health, and food-safety regulators, in which Codex Alimentarius, OIE, and other NGO’s play an important intermediary role. The finally agreed benchmark is therefore per definition dated. Accordingly, the gold standard is not a perfect and most accurate method [233]. On the contrary, reference methods are frequently challenged. A well-known example is the several orders of magnitude of discrepancy between the viable bacterial plate count, the gold standard, and the microscopic count of bacteria [234]. Another example is the gold standard for subtyping foodborne bacteria for over two decades, namely pulsed-field gel electrophoresis (PFGE), while other well-validated superior methods are available [235]. Consequently, improved performance by an innovative assay is frustrated by comparison with the benchmark as it will give *a priori* worse relative SE, SP, and AC.

Compared to a classic, reference ELISA using colorimetry of an enzyme-generated chromophore, an alternative method using fluorophores provides a signal order of magnitude more linear. Consequently, responses outside the linear range of a chromophoric ELISA do not congruent and inside this range, it is probably with another steepness. This is not acceptable in validation programs and not for accreditation bodies either (dr. Bergwerff, unpublished).

Another diagnostic validation hurdle is the multitude of information that a (real-time) multiplex assay produces, while reference methods are typically traditional, single end-point measurements. For example, indirect antigen *Salmonella enterica* ELISAs produce a result per sample which is a resultant of immunoglobulins reacting to (a mixture of) immobilized O-antigens in a single well. The O-antigen mixture represents serogroups B, C1 and in some versions supplemented with serogroups D and E [156]. Multiplex assays can provide information for each serogroup independently. In a BBA, each serogroup is represented, for example, by a single microsphere. However, the reaction dynamics and kinetics on a coated surface of a microsphere compared to those in a static well, even when agitated, are significantly different. The sum of fluorescent responses of each microsphere does not coincide with the resultant colorimetric response in a well using an identical sample (dr. Bergwerff, unpublished).

Despite good repeatability and reproducibility performances of an assay, special attention should be given to so-called assay drift within or between runs, and within and between days/weeks [236]. This comes often unnoticed in microbiological assays as, unlike most chemical assays, internal standards in food microbiology are difficult to define and/or are not stable (under assay conditions and/or by e.g., freeze-thawing cycles). For this and other reasons, when a test passed a validation program and used to determine contaminations in animals and/or food products, its validity has to be verified ongoingly in a proficiency test or “ring test”, although this test also assesses the quality of laboratory and operational factors [229].

### 3.2. Predictive Values

A result of an indirect antigen test is not an endpoint indicator of an infection or contamination of an individual animal or a food product as explained above. An endpoint is acquired by repeated analyses (monitoring), or evaluation of a set of results reflecting a herd/flock or batch (eggs). However, evaluation of the contamination level of batches or herds/flocks based on tests applied to single products or individual animals is complex. An aspect that is regularly out of view of inspectors and analysts is the prevalence (persistence) and incidence (spreading) of some pathogens, in particular when these are very low. Prevalence depends often on a geographical region. *Trichinella* spp. in swine raised under (un)controlled conditions is in the EU virtually 0% (0–0.0003% in the period 2014–2018) with few human cases [18]. All positive cases involved pigs which were raised under non-controlled housing conditions, in particular in the Member States Croatia (58 per 9.5 10^5^), France (5 per 6.0 10^5^), Italy (8 per 1.6 10^5^), Poland (39 per 22.7 10^6^), Romania (134 per 2.5 10^5^), Spain (5 per 27.7 10^6^) [18]. *Salmonella* prevalence in swine herds in Scandinavian countries is near 0% as well [237]. A low prevalence requires other assay characteristics. After all, the outcome of all (obligatory) tests performed on pathogens with a negligible occurrence in a population is prevalently “negative”. In that case, assay sensitivity is of less importance, whereas, reciprocally, assay specificity is gaining importance, i.e., rate of false positives. A false-positive result can have namely dramatic effects, such as the condemning of herds, closure of farms (also those near the false-positive herd), and even the closing of borders for the international trade of animals and their products. In other words, the negative and positive predictive value of an assay is of utmost importance given the prevalence of a pathogenic MO.

There is a relationship between sensitivity and specificity through the threshold of the assay so that the chance of false-positive results can be reduced. A consequence of such reduction is that the false-negative rate increases concordantly. However, in contrast to human testing, animals are grouped in herds and are sampled and tested in clusters. Assessment of a cluster will normalize the results and blot out the impact of false negatives and false positives. On basis of prevalence and desired confidence level, statistics will help an inspector to determine sample size and how many samples are ‘allowed’ to be positive in a cluster before it has to be considered positive. The interested reader is referred to e.g., [110,111] for further information.

## 4. Example: *Salmonella* Detection in The Pork-Production Chain

To illustrate the methodological challenges of pathogen screening to warrant food safety, the monitoring of *Salmonella* in pork as part of intervention and eradication programs is discussed here. If hygiene is assured further down-stream, intervention is most successful in the primary sector of the pork production chain. The intervention relies greatly on testing of the pathogen, and timely and adequate acting upon a positive finding. However, when choosing the wrong strategy, the most excellent analytical method will deliver a wrong conclusion on a carrier status as explained below.

Ideally, bacteriological examination of feces from multiple animals will deliver an accurate infection status of a pig herd at the moment of sampling. Positive isolation and confirmation of *Salmonella* will leave little doubt to its presence in the sampled animal. However, a negative bacteriological test result should be interpreted prudently. After oral exposure, *Salmonella* migrates rapidly to the caecum and, consequently, it can become undetected by bacteriological investigations of feces. Furthermore, competitive saprophytic microorganisms, such as coliforms, dominate *Salmonella* which usually becomes injured and therefore problematic to culture (*cf*. Section 2.3.3.1). Consequently, fecal *Salmonella enterica* shedding is usually intermittent, at low numbers, and observed predominantly in the first half of the fattening period [238,239,240]. A delicate consequence is that when non-shedders are slaughtered non-hygienically, carcasses can be a source of contamination in the slaughter line and further down the food-chain.

Direct antigen detection of *Salmonella* is thus difficult in subclinically infected herds, which are much more frequently encountered than herds with clinical signs [240]. The diagnostic sensitivity of microbiological culture methods of fecal samples, which are considered reference methods, is relatively low in a practical setting, namely 50–60% compared to that of the fecal samples collected at the abattoir [241]. As a result, the negative predictive value of direct antigen screening of herds is poor and the correlation of bacteriological results with the risk status of the herd is weak [242,243].

On the other hand, anti-*Salmonella* antibodies persist beyond the time of infection and indirect antigen testing reveals a historical exposure per definition. Serological data do thus not correlate closely to the apparent bacteriological burden at the time of sampling [244]. While fecal *Salmonella* (intermittent) shedding is observed in the first half, seroconversion occurs generally during the last third of the fattening, i.e., the pre-harvest period [238,239]. However, a *Salmonella* infection during transport to an abattoir or in the lairage does not result in a timely seropositive reaction, as there is an insufficient interval to induce a detectable immune response before slaughter. Maternal anti-*Salmonella* antibodies, for example, persist more than eight weeks in the off-spring and cannot be distinguished from the pig’s own antibodies in that period. This fact is of importance as most piglets are weaned just three weeks after birth. They arrive in new pens/groups and an introduction of a new *Salmonella* infection cannot be distinguished on serological data until eight weeks of age [238].

Despite these contraindications and the apparent asynchronous bacterial infection and serological status [244], the prevalence of seropositive swine on herd level at slaughter correlates significantly with *Salmonella* in rectal contents and mesenterial lymph nodes [245,246]. Actually, indirect antigen assays are considered to have a higher diagnostic sensitivity than direct antigen tests [240,247], in particular as these tests can reveal latent carriers or intermittent shedders. The *antemortem* serological status of the herd gives thus a good estimate of the risk of *Salmonella enterica* spp. in pig products [245,248]. Despite a conversion window of days for each animal, the varying individual infection onsets on a herd level, varying individual seroconversion intervals, and varying immune response intensities will give a rather reliable overall indirect antigen testing outcome. Pig farms have to acquire a *Salmonella* status classification based on direct and on indirect antigen monitoring regularly (three-monthly in some countries). Classification of herds is in accordance with a certain percentage of positive animals and determines whether farms are required to increase prevention activities and reduce *Salmonella* prevalence [249,250]. This successful system was developed and introduced by Denmark in the 1990s [77,251].

This example of screening does not advertise exclusive indirect antigen testing. Feed, water, and environment are integrally part of monitoring systems to maintain biosecurity and they are commonly investigated using direct antigen tests, including NASB tests.

## 5. General Discussion and Conclusions

### 5.1. Responsive and Smart Monitoring and Control

Foodborne infections and food spoilage are persistent global problems that keep on giving analytical microbiology a pivotal role in prevention, intervention, and outbreak control. The population of vulnerable people grow vastly, and eating habits, agricultural and manufacturing practices, and climate change. *Ergo*, there is a growing “opportunity” for unfamiliar pathogens to emerge and for familiar pathogens to re-emerge. Irrefutably, a firm need to assess and prevent infections and contaminations in the food-chain will hold on.

Methods, design of food inspection, and intervention regimes stem from the end of the 19th and the beginning of the 20th century. After a long standstill since the enforcement of the first general food laws, food inspection and control of food hazards was scrutinized and modernization advocated [252,253]. As we are continuously striving “to die young in life as late as possible” (after late Prof. Bob Kroes, Utrecht University, Utrecht, The Netherlands [254]), it became clear that new ways of thinking about how to deliver improved monitoring to secure food-safety are required. The one-health concept (OH), although the term was already coined in 1964, emerged from this. It advertises to secure food safety starting at the farm into the chain by warranting pathogen-free feed, water, environment, and healthy animals [255]. It is in fact an inter-professional area linking the human-animal-environmental health triad on which was elaborated by Prof. Frans van Knapen and co-workers (Utrecht University, Utrecht, The Netherlands) since the end of the last century [256]. As a result, the EU is the first supranational government worldwide implying the “farm-to-fork” approach and to require visual-only inspection for all swine herds slaughtered meeting certain epidemiological and animal rearing conditions [257]. The system obliges producers to provide so-called food-chain information (FCI), including data revealing herd’s health status and zoonotic risks. The status, i.e., herd risk level, is based for a great part on the outcomes of indirect antigen (serological) monitoring, including that of *Mycobacterium avium*, *Toxoplasma gondii*, and *Salmonella*. Apparently, as in inspection and control systems in non-food sectors, big data analysis gets a foothold in the food-chain as well. In fact, the first signs of involvement of blockchain technology fueled by supermarkets are visible.

In the scope of this review, this reorientation on keeping food free from contamination means that indirect antigen methods will survive despite upcoming other measuring principles, such as genotyping methods. The poor diagnostic sensitivity of current NASB methods, as highlighted above, makes them in several cases unfit for routine livestock screening in novel responsive, as opposed to inflexible bureaucratic, inspection systems. On the other hand, environmental monitoring, which is essential in an OH approach, is best served with the availability of multi-analyte direct antigen detection systems with a wide range of applicability, which also includes NASB methodology. It should be able to deliver data before the product is transported/delivered to the next link in the food-chain, which means not rapid *per se*.

Besides having mitigation strategies in place at crucial points in the chain, modernization also requires risk-based sampling, monitoring, and effective, targeted surveillance. After all, *Salmonella*, for example, is more of a problem in West-Europe than in North-Europe, while *Taenia saginata* and *Trichinella spiralis* have a higher prevalence in East-Europe than in the rest of the continent.

### 5.2. Prediction, Indicators, and Prevention of Sherlock’s Holmes Statistics

One of the strategies is controlling biohazards using predictive microbiology to quantify microbial ecology (of foods) of bacteria [258]. The use of loggers of, for example, temperature, retrospective analysis of data supports the prediction of the contamination status of food [259]. This approach may lead to, net, less sampling, and less laboratory MO-testing, but testing at higher quality and more to-the-point instead.

Actually, there is also a statistical reason for reconsideration of our conventional monitoring systems. An ever-increasing higher security level demanded by the consumer is obstructed by what is called “Sherlock’s statistics”. A low prevalence level and an increasing confidence level require more and more samples to deliver the requested reliability. For example, at a 20% prevalence in a population or batch, only 11, 14, or 21 samples are needed to reach a confidence level of 90%, 95%, and 99%, respectively. However, given a prevalence of 0.1%, 2302, 2995, and 4603 samples, respectively, are necessary to meet the same confidence levels [260]. Actually, in *Trichinella spiralis* monitoring programs each and every pig carcass has to be investigated by a so-called artificial digestion method by law to meet the required highest confidence level [261] at extremely low prevalence in most countries [262].

To make *Trichinella spiralis* monitoring even more complicated, the sensitivity of the digestion method can be, depending on the burden, depending on the stage of infection in the animal and whether samples are pooled, as low as 40% [159,160]. An immune response-based method may deliver more reliable infection status data not of an individual carcass but a group [63,159]. In the case of a low prevalence of a pathogen with a serious health threat, such as *Trichinella spiralis*, the solution can be monitoring of antibodies in a herd against a pathogen occurring in the environment with a higher prevalence as well. Like *Trichinella* spp., *Toxoplasma gondii* is an indicator of contact with the environment. Infection routes are shared at which those for *Toxoplasma* are wider and more extensive than those for *Trichinella*. In other words, *Toxoplasma* infections are an excellent indicator of farm hygiene, contact with the environment, and good practices (GAP, GHP). Finding a *Toxoplasma*-positive result indicates increased risk for several other public health-threatening zoonoses, including *Trichinella* [159]. Humoral responses to these bioagents are dynamically independent and co-infections do not influence each other [263] so that screening anti-*Toxoplasma* antibodies can support and improve pork monitoring and control programs e.g., *Trichinella* spp. [159]. It is an intriguing strategy that should be elaborated on for all farm animals and all relevant MOs if pathogens-free has to be warranted at a low prevalence and at a high confidence level.

### 5.3. The Weak Link

A chain is no stronger than its weakest link. Whatever effort upstream in the food-chain, threats are clear and present downstream in mass kitchens and domestic situations. Factually, the relative contribution of contaminations in the third and quaternary sectors (Figure 2) are already causing the majority of foodborne infections with norovirus as the champion of all causative bioagents. The relative contribution in this last part of the food-chain will increase as an intervention in the (pre-)harvesting and processing phases become even more effective. Changing eating habits, poor personal and kitchen hygiene, pets, pests, etc. contribute substantially to all foodborne infections. *Yersinia enterocolitica* and, in particular, *Listeria monocytogenes* seem to have adapted themselves to modern food production and preparation. They grow at refrigerator temperatures and are persistent unwanted guests in processing facilities and households. Is there a more prominent role for food producers, authorities, and/or dietitians to educate the consumer [42,43]? Can kitchen equipment be engineered more smartly so that the chance of contamination is reduced and in such a way that cleaning every corner and edge is easier?

Currently, spoilage/freshness indicators are increasingly applied to food packages (see [264] for an overview) in which IA-based methodology is also used. However, only a few fail-safe, rapid methods, as it were modern food-tasters like at the courts of kings and emperors, are available to laypersons to check the safety of food and adequate hygiene in institutional kitchens or at home. Here, assay developers find a new, growing market for cheap, stable IA methods. LFD technology or alike platforms seem to have a very good starting position for this market.

### 5.4. Fool’s Gold?

The need to analyze multiple MOs simultaneously in a virtually indefinite number of different analytical complex matrices is outweighing the requirement for more sensitivity and shorter time-to-results. An animal or food product, let alone a herd or a batch of products, is not a potential carrier of a single species, (sub)subspecies, but can be infected/contaminated with quite some bacteria, bacterial toxins, parasites, and viruses (*cf*. Figure 1). It says that there is a preference, at least in field laboratories, for protocols enabling multi-analyte and high-throughput screening more than for methods providing more speed and sensitivity.

*Sensu lato*, magnificent speed-of-analysis, and impressive analytical sensitivities are demonstrated, and almost all developers claim that their innovation will replace laborious, cost-inefficient, time-consuming traditional methods. A majority of these developments show indeed excellent qualitative or quantitative analytical advancements, but they fail to report transparently the evaluations of diagnostic sensitivity and diagnostic specificity. Sophisticated, sensitive NASB techniques will namely most likely give false-negative feces results as e.g., *Salmonella* hides intracellularly in lymph nodes in swine of three months and older [240] (*cf*. Section 4). Moreover, many improvements are specialistic as well, they are described for only one or a small set of MOs in one or a few analytical matrices. These investigated matrices are often spiked and not naturally infected for the development/validation/performance studies. Many microbiologists can explain to these developers that there is a world of difference between spiked samples and samples from natural infections.

Developments giving means to simple multiplexing analysis have considerable market potential. They have an unexpected advantage which can be “fool’s gold” or a “driver” (*cf*. Table 5). Up to 22% of acute hospitalized gastroenteritis cases are caused by two or more pathogenic MOs [265,266]. Food products may be contaminated with more than one pathogen. In fact, more than one *Salmonella enterica* serovar in poultry and swine, and curli- and non-curli-producing *Escherichia coli* O157 strains in beef were observed regularly in the author’s laboratory (dr. Bergwerff, unpublished results).

An ultimate solution seems a holistic approach using a chip coated with probes for more than 12,000 archaea, bacteria, fungi, protozoa, and viruses suggested for veterinary use as well [267]. The analytical sensitivity of this technology is approximately 100–1000 genome copies but it does not deliver verifiable quantitative data. Of interest here is how the specificity of the multiplexing chip is secured. An increasing number of probes will also swell the chance of (weak) a-specific binding and thus a good chance that one or more of the 12,000 probes give a false-positive result. It will affect the overall false-positive rate. But even true positive responses do not imply safety relevancy per se without quantitative data of the detected MOs. In some cases, for example, PRRS in swine, the impact of a positive sample can be dramatic, *viz*. immediate closure and isolation of the farm from which the sample was collected. Danish field laboratories for example demand therefore a diagnostic specificity for PRRS near 100%.

Routine laboratories need reliable universal methodologies to acquire all relevant answers with a single technology. The desire arises not only from a cost-efficiency point of view but also for other reasons, such as comparability of results, participation in proficiency programs with a method which is considered not to be unusual.

### 5.5. Validity and Comparability of Results

The higher specificity of genotyping methods is a clear advantage over IA methods. Although IGs and AGs react with a relatively high degree of specificity in IA assays, other MOs can share (similar) antigenic structures with the target organism giving false-positive outcomes. Therefore, direct and indirect antigen tests are regarded as presumptive. A positive result in a direct IA antigen test has to be confirmed through e.g., conventional culture procedures. A positive outcome from a single indirect antigen test is considered without much value. Reliability of serology is obtained by repeated sampling (monitoring) or analysis of a series of random samples to assess a group (herd or flock). Having noted this, the specificity of NASB assays can be too strict as demonstrated by their failure to pick up SARS-CoV-2 mutants [222].

Analytical food microbiology seems to be based increasingly on the determination of a genetic code [268] of in particular bacteria and viruses. Despite the high analytical sensitivity of these techniques, their diagnostic sensitivity has to be scrutinized carefully. After all, a test has to show e.g., the absence of a disease-causative agent in a 25 g sample, or in 1 to 4 g tissue in the case of some parasites and has to make a difference between viable and non-viable pathogens. Isolation of a few bacterial cells from a 25 g sample to deliver a portion of only a few microliters or less is still practicably impossible on a routine basis. Even IMS does not deliver sufficient recoveries in complex food matrices. To mitigate these analytical hurdles, sample preparation, such as inoculation of a broth and culturing of the bacterium or virus, is needed and this will delay inherently the time-to-result. This sample preparation is often suitable for NASB and IA methods so that a final decision on an analytical approach is determined on criteria listed in Table 4 and Table 5.

A difficulty with IA-based and NASB techniques is that they do not uniformly express results. The optical density of an ELISA result is not comparable with the dynamics of a fluorescent signal in a BBA assay or with a cycle threshold (CT) number in a qPCR method. *Salmonella* surveillance programs rely commonly on indirect antigen ELISA analyses and use thresholds expressed in OD values. It prevents the introduction of for example BBA methodology showing other, from the authors’ point of view much better, signal dynamics. This incomparability is also apparent from the difficulty to verify a result produced by an alternative method against legislative norms expressed in, for example, colony forming units per mass or volume unity (Table 6). The norm for *Campylobacter* in broiler meat, 1000 CFU/g (Table 2), cannot easily be verified with a PCR-based method, or with any other non-traditional method for that matter.

Despite all advancements, the ideal method (Table 4) does not exist. For a new alternative method to get implemented it has to have a profile that provides maximum end-user satisfaction in combination with the greatest possible differentiation from technologies already in use in his laboratory while results are comparable and stoichiometric (Table 5).

### 5.6. Weighing the Investment in New Methodology

In a field laboratory, the decision-maker has little attention to the scientific incentives for the acquisition of new technology. He/she assumes that the scientific performance has been approved by her/his laboratory co-workers. In her/his final decision, she/he is interested almost exclusively in the continuity of the laboratory and in overall costs. When asked, reduction in TTR is not as important as cost reduction in many routine laboratories (dr. Bergwerff, unpublished). When a big test producer with its headquarters in France, started offering a 24 h instead of a 48 h *Salmonella* test, only 10–15% of the users converted to the new test. This was because the majority of *Salmonella* tests are for screening/monitoring purposes [268] in which case a difference of a day does not outweigh the higher costs of a faster method.

Of all pathogen tests used worldwide, almost 50% gauge *Salmonella* in processed food and meat (each approximately 40% of the total market). The majority of these tests are traditional plating or petrifilm^TM^ tests in Europe and North America while genotyping assays are more popular in Asia [268]. Although the balance of types of tests will change over time, it is probably disappointing for the novel assay developer. Routine laboratories are reluctant to switch to new technologies as tolerance for failures -read economic impact on the organization- is very limited. After all, the margins of the added value of food are very small and the cost reduction of an alternative testing technology must be very convincing. The price, not costs, for a *Salmonella* test, varies between less than €1 (indirect antigen) to approximately €4 (direct antigen) in field laboratories Europe-wide (dr. Bergwerff, unpublished). The price must not only deliver profit e.g., new investments but should also cover all costs for the laboratory, including the costs of purchasing the test, overhead, (training of) personnel, sample handling-time, sample collection, storage, and preparation, laboratory infrastructure (footprint), depreciation of equipment, operation failure (down-time), false-result rates, (equipment) maintenance, etc.

Concerning (frequency of) down-time, in several instruments, parts, such as sensor chips, have to be replaced or regenerated for each analysis cycle. Without losing critical analysis capacity, regeneration of sensor surface was up to 300 cycles in a *Salmonella* indirect SPR immunosensor test [188]. A sensor can bleed or the regeneration conditions degrade and inactivate the ligand. Actually, many publications do not report the stability of the prepared measuring interface/instrument. Replacing sensor chips or other hardware, also in the case of single-use, is lost time and not attractive for a routine laboratory commonly receiving hundreds to thousands of samples daily. The reader notices here a subtle difference between TTR and throughput: A slow high-throughput method can be more efficient than the fastest (single analyte) test.

Down-time can also be caused by unforeseen factors. Meat drip is the analytical matrix of choice in national monitoring programs of some countries and is collected by butchers or inspectors (not laboratory personnel). As muscle has more value than fat tissue, it is tempting to collect samples with high-fat content. These samples give not only measurement interference, but also clog tubing, needles, and pipette tips in liquid handlers and other instruments.

### 5.7. Bioprepology

The fat issue in meat drip is a bridge to an important but often neglected subject: sampling, sample handling, and sample preparation. Although Prof. Chris Elliott (Queen’s University Belfast, Belfast, UK) not exclusively emphasizes the subject, he states that “food analysis is all about sample treatment” and coined therefore the term: “bioprepology”. The term expresses not only the gravity of a crucial step in any food analysis procedure, and in fact, in any life science method, it also legitimizes the existence of a separate, specialistic scientific discipline. The holy grail in food analysis is namely a universal and minimal sample pre-treatment for a great variety of (complex) matrices, while the resulting test portion still reflects integrally the animal, plant, or food product from which the sample was taken. In many cases, especially direct testing of MOs, sample treatment is a bottleneck for fast, inexpensive, and easy-to-use (IA) assays.

When sampling a contaminated carcass, one should realize that a swab may give a false-negative result. First of all, MOs are not homogeneously distributed over a carcass, and swabbing a too small surface on a single, improper location may give a wrongful negative result. Therefore, surface areas for swabbing are regulated. Secondly, after culling and by stressing the bacterium, *Campylobacter* retracts from fecal spillage into the skin by adhering and invading epithelial cells [269]. But even with a proper sample, *Campylobacter* may remain unnoticed when it has converted into a non-culturable coccoid state (VBNC) [269].

### 5.8. Conclusions and Messages

- A great part of the *ante* and *postmortem* monitoring comprises indirect antigen assays gauging specific antibodies in serum, meat juice, and oral fluids.
- When (intracellularly) hidden or low body burden, IA assays outperform other analytical techniques, including NASB methods.
- IA assays offer a chief advantage over NASB assays: they can detect acellular biomolecules, including toxins, uncovering a (past) infection.
- The largest part of analyses worldwide involves the *ante*- and *post-mortem* monitoring of MOs in the (pre-)harvesting phase of the food-chain. Almost 50% of all tests involve measuring *Salmonella*. IMS plays an important role.
- Whatever analytical sensitivity, analytical specificity, and other test characteristics, the applied assay should fit the purpose while it is clear when and where it is used in the food-chain.
- Novel methods should be presented with data from field samples, not from spiked or polished reference samples exclusively.
- Integrated chain control and One Health principles in combination with risk-based sampling are imperative to combat effectively current and (re-)emerging pathogens while increasing the safety level of food.
- Successful intervention on the guidance of (environmental) monitoring will also protect (families of) farmers and food-workers as a good health and safety practice.
- The need for more speed and sensitivity is modest and not prominent in field laboratories, albeit results within a working shift are highly desired.
- Mutual comparison of results produced by a gamut of alternative analysis systems and comparison with reference methods is an unsolved challenge.
- In the case of group assessment, routine laboratories prefer high diagnostic specificity, multiplexing, and high throughput, but convincing low all-inclusive costs even more.
- All steps between the decision to sample and conversion of a sample into a test portion need continuous and careful attention or the analysis result becomes less reliable or even worthless. Sampling, sample treatment, and sample processing are of cardinal importance.
- In spite of the numerous innovative techniques that evolved over the last decades, only a few have been authorized for screening, monitoring, and control programs.
- The food analysis field is conservative for several understandable reasons, not only because of financial risks. Routine laboratories are bound to accreditation and providing results as if generated by reference methods.

## 6. Future Perspectives

The outer limits of food science including food microbiology and in particular food microbiological screening techniques are not clear. Whether direct and indirect antigen assays will keep claiming an important role in securing food safety as they did for decades, is uncertain. Nevertheless, there is a mindful trend for methods offering ease-to-use, in situ results, full risk assessment (multiplex), and high sample throughput in a true culture-independent way. What is the heuristic direction towards the best technology in the analysis of foodborne MOs? A glance at the end-user requirements may help:

(1) Test performances that are compliant with local, national, and international (ISO) quality standards.
(2) Enabling the use of (relatively) easily available, preferably non-invasive, samples, such as egg, feces, hair/feathers, saliva/mucus, urine.
(3) Able to analyze simultaneously different types of analytes, such as cells, oligo-/polynucleotides, oligo-/polypeptides, organic metabolites, toxins, in a single run.
(4) Easy-to-use or automated platform demanding minimal user involvement.
(5) Giving accurate results instantaneously (i.e., within 10 min in a PoC situation or otherwise in a work shift).
(6) At low costs, while,
(7) Using a robust, reliable portable multiplex point-of-care testing device (xPOCT) and reagents that have a long shelf-life at ambient temperatures, and which are easily disposed of after use.

New technologies, in particular those based on nucleic acid sequences, seem to give a peek of the future: detection and identification of even the fastidious and/or non-cultivable bioagents in a rapid, cheap, and reliable way. The advent of for example (droplet) digital PCR techniques [270], which are less affected by enzyme inhibitors in complex analytical matrices such as food or feces [220], may supersede IA techniques as soon as they are also able to deliver good diagnostic specificity and diagnostic sensitivity. The technique also promises a possibility to quantify bacteria reliably so that it may replace the counting of colonies in plating methods.

Considering the last half-century, the design and synthesis of antigens, of (derivatives of) immunoglobulins, and chromophores, and fluorophores has progressed slowly compared to NASB technology. The promise of immunosensors in food microbiology has not been fulfilled (yet) despite the investments and research efforts put in the development of this type of instrument. In the end, the decision to change testing systems is determined by legislative restrictions, (international) trade agreements, and costs of sample collection to result in interpretation and reporting. The food analysis market is a very conservative world. In the practice of an ordinary (commercial) food-safety laboratory, applied methods evolved unhurried.

On-site determination of different analytes from a single specimen in a single run (xPOCT), is gaining increasing attention in clinical diagnostics [208]. This development will certainly find its way to surveillance systems in the austere environments of the food-chain like many other advancements in human medical settings did before. After all, intervention at the farm is the best approach to prevent contamination down the production line.

An interesting development is the detection of volatile and non-volatile biomarkers for pathogens that ferment materials in or on animals, feed, food products, or plants. Screening of such indicators may support and even improve surveillance programs. E-noses or dedicated miniaturized MS devices, for example, can sample air and select suspect farms and processing plants for further confirmatory analyses. Such devices can sample livestock houses continuously and on multiple locations and activate alarms and automatic sampling through internet-of-things (IoT). It will relieve laboratory testing while making it more effective when alarms have to be confirmed. The implementation of machine-learning is perhaps to come. It may help to integrate seamlessly methods in various contexts and applications, which are producing uninterrupted unfathomable high volumes of raw analysis data and gathering other information in the food-chain.

Ultimately, we strive for utopian sci-fi methods: technology like the Star-Trek tricorder device [271] which gives non-invasively, instantaneous, and easy-to-interpret results and which can be operated by anyone not only by a skilled person like Doctor Leonard McCoy!

## Figures and Tables

**Table 1:** Confirmed human cases associated with foodborne pathogens in EU member states and associated countries in 2019 [19].

Pathogenic Micro-Organism	Confirmed Human Cases (Number)	Case Fatality (%)
Campylobacter	220,682	0.03
Salmonella	87,923	0.22
Shiga toxin-producing *E. coli* (STEC)	7775	0.21
Yersinia	6961	0.05
Listeria	2621	17.6

**Table 2:** Steps and phases in an analytical procedure determining a bacterial pathogen which can cause foodborne infections (adapted from [109]). These steps involve prevalently those for direct antigen analyses.

Step/Phase	Note
To investigate	Water: environment, processing water, drinking water (for animals or to prepare a meal). Environment: farm (including wild animals and insects in its surroundings), processing plant, abattoir, butcher, greengrocer, kitchen, etc. Pre-products: carcass, ingredients (herbs), etc. Products: fruit, meat, sliced vegetables, ready-to-eat, salads, etc.
Sampling	Sample quality and size should reflect what is investigated (food, flock, farm, herd, retailer, kitchen, etc.)
Transport	Identifiable at all times, properly cooled, and with no risk of cross-contamination
Sample treatment	Acclimatization Homogenize if required (as a matter of fact, homogenization is a specialism in itself)
Pre-enrichment	Resuscitate and proliferate bacteria to determine even low numbers
Selective enrichment	Proliferate the aimed pathogen exclusively
Culture evaluation	Gauge selective culture by assessing color, smell, turbidity and microscopical investigation, Gram staining, etc.
Analysis	Traditional plating, agglutination, enzymic assays, LFD, (IMS) ELISA, apta-/geno-/immune-/phagosensors, LoaC, LoaD, nucleic acid sequence assays
Verification	Confirmation and identification

**Table 3:** A selection of norms for pathogenic MOs in some food products [3]. The criteria apply at different stages in the food-chain and are associated with a sampling plan and with certified ISO methods not indicated or included in this table.

Pathogen/Predictor	Food Product/Matrix	Norm
*Listeria monocytogenes*	Ready-to-eat ^a^	Absent in 25 g
*Salmonella*	Cheese, butter, cream from raw or non-pasteurized milk	Absent in 25 g
*Salmonella*	Meat products intended to be eaten raw	Absent in 25 g
*Salmonella*	Meat preparations intended to be eaten cooked ^b^	Absent in 10 g
Staphylococcal enterotoxins	Cheeses, milk powder, and whey powder	not detected in 25 g ^c^
Enterobacteriaceae	Egg products	10 or 100 CFU/mL or CFU/g
*Campylobacter* spp.	Carcasses of broilers	1000 CFU/g
STEC O157, O26, O111, O103, O145 and O104:H4	sprouts	not detected in 25 g ^c^

^a^ Exceptions of the norm if ready-to-eat food is not intended for infants or special medical purposes then 100 CFU/g. ^b^ If made from poultry, then *Salmonella* should absent in 25 g. ^c^ “not detected in 25 g” using a specified ISO method: implying an LoD not necessarily meaning “absent in 25 g”.

**Table 4:** Typical end-user requirements for assays that determine MOs in the food-chain. The order of listed requirements is arbitrary.

Requirement	Note
Low cost	Test outcome should provide sufficient information to make a contemplated decision at costs that balances investments and consequences. Costs include overhead, maintenance, personnel, laboratory footprint, disposables, etc.
One-pot reaction	Complete preparation and processing in a single vial/tube. No addition of reagents required (~ easy-to-use).
Range of application/ operation	Versatile: *sensu lato* applicable to fresh and processed food matrices, and/or for samples from feed, plants, and animals. Suitable for all relevant bioagents.
Multi-analyte/multiplex	Able to determine multiple targets in a single analysis run.
High-throughput	Multiple samples run simultaneously.
(relative) accuracy	Free from systematic and random errors.
Reproducible (precise)	No or insignificant variation (in-)between runs, days, machines, analysts, etc.
Repeatable	No or insignificant variation between laboratories, batches, lots.
(Relative) specificity	100% specific compared to the reference method (no false positives); able to distinguish at the strain level.
(Relative) sensitivity	100% sensitive compared to the reference method (no false negatives); able to detect (viable) MO at required sensitivity ^a^. Excellent signal-to-noise ratio.
Confirmation of analyte	Juridical problems arise when the identity of the analyte is not confirmed. There should be a reliable answer to the question: what is the degree of certainty of the identity of the aimed target giving the result?
Automable	A stand-alone instrument with limited hands-on time. The frequency of personnel attention is low.
Portable/Point-of-care (PoC)	Ambulant, performing analysis in/at/on-line of the food-chain with interpretable results available on site.
User-friendly and fail-safe	Easy-to-use, performed by non-skilled personnel, preventing facultative and inherent errors. In other words, the assay is rugged and gives the same result even when test conditions such as incubation times, operator, pH, reagent concentrations, temperature, etc. vary. This also includes safe to use.
Easy to interpret	Analysis data give a transparent, unambiguous, and understandable result
Instantaneous result	Results available real- or near-time. Although not meaning the same, time needed for the whole analytical process from sample collection to result (TTR) should be short. In practice, TTR can imply a result within a working day/before the next phase in the food-chain.
No sample treatment	The test portion is obtained directly from the laboratory sample.
Robust	Reliable operation under e.g., humid, varying temperatures, bumping conditions. No drift/long-term stability.

^a^ Required sensitivity: Detection level at which the MO does not pose a health risk or at the legislative norm.

**Table 5:** Relevance versus differentiation criteria of (innovative, alternative) analytical food microbiology methods for routine laboratories. The various method parameters that make up the overall profile are separated into four categories that reflect their relative attractiveness to users and differentiation from assays that are already in use. The completion of the quadrants is empiric and the weight of criteria may vary between readers.

*High*	Antes ^a^	Drivers ^b^
**Relevance**	Equivalent accuracy to reference methods. Relevant matrices (farm, feed, abattoir, food, and beverage). Required validation(s), such as AFNOR, UKAS. Reference labs for new users to contact. Robust system.Information handling—LIMS connectivity Security of access and data (CFR 21 pt. 11 etc.). Stoichiometric results compared to the reference method. CE Mark	Lower overall costs than other tests. Multiplexing relevant combinations of Mos. Time-to-result in a working shift. Fits factory sampling and laboratory routines. Closed system: sample in-result out No enrichment or otherwise universal enrichment broth. Can detect, identify and confirm within the same test. Smaller footprint than reference tests. Flexible number of tests per day. Possibility to enumerate. Single platform for direct and indirect antigen testing.
***Low***	**Neutrals ^c^**	**Fool’s Gold ^d^**
	Low waste. Fashionable design. Product source.	Fast time-to-result in cases where time is not of the essence (e.g., farm surveillance). High sensitivity in cases where the normal.
	***Low*** **Differentiation** ***High***	

^a^ Antes, important but provided by all methods; ^b^ Drivers, important and which highly differentiate from those of reference methods; ^c^ Neutrals, irrelevant; ^d^ Fool’s Gold, distinctive, but which do not convince users to switch.

**Table 6:** Comparison of characteristics of direct and indirect antigen methods (*cf*. Table 4). The table only lists those items which differ significantly.

Traditional (Direct Antigen)	Alternative (Indirect Antigen)
Laboratory-bound	Point-of-care possible
Intensive material use	Relative expensive material
TTR long	TTR usually short
Reproducibility moderate	Reproducibility mostly better
Almost no instruments	Needs more devices
Difficult to automate	Possibilities to automate

## Abbreviations

AB	antibody
AC	(relative) accuracy
AFNOR	Association Française de Normalisation
AG	antigen
AgBP	antigen-binding protein
AOAC-RI	Association of Official Agricultural Chemists Research Institute
ATP	adenosine triphosphate
BBA	bead-based assay
BSE	*bovine* spongiform encephalopathy
CE	Conformité Européenne (standard mark)
CFU	colony-forming units
CPA	cross-priming amplification
CPS	capsular polysaccharides
CSF	classic swine fever
CT	cycle threshold (as in qPCR)
DALY	disability-adjusted life year
DES	diethylstilbestrol
DIVA	differentiating vaccinated from infected animals
EF	extracellular factor (of *Streptococcus suis*)
EFSA	European Food Safety Authority
EIA	enzyme immunoassay
ELFA	enzyme-linked immunofluorescent assay
ELISA	enzyme-linked immunosorbent assay
ESI	electrospray ionization
FCI	food-chain information
FSO	food-safety objective
GC	gas-chromatography
GAP	good agricultural practice
GHP	good hygiene practice
GMP	good manufacturing practice
GVP	good veterinary practice
HACCP	hazard analysis critical control points
HAV	hepatitis A virus
HEV	hepatitis E virus
HRP	horseradish peroxidase
IA	immunoaffinity
ICT	immunochromatographic test
IG	immunoglobulin
IgA	immunoglobulin class A
IgG	immunoglobulin class G
IgM	immunoglobulin class M
IMS	immunomagnetic separation
iPCR	immuno-PCR
iqPCR	real-time immunoquantative PCR
IR	infra-red
ISO	International Organization for Standardization
LAMP	loop-mediated isothermal amplification
LFIA	lateral flow immunoassay
LIMS	laboratory information management system
LFD	lateral flow device
LoaC	lab-on-a-chip
LoaD	lab-on-a-disc
LOD	limit-of-detection
LPS	lipopolysaccharides
MAB	monoclonal antibody
MALDI-TOF	matrix-assisted laser-desorption ionization time-of-flight
MIP	molecular imprinted polymer
MO	micro-organism
MS	mass spectrometry
NASB	nucleic acid sequence-based
NASBA	nucleic acid sequence-based amplification
NGO	non-governmental organization
NMR	nuclear magnetic resonance
NPV	negative predictive value
OD	optical density
OH	one health
OIE	Office International des Epizooties (World Organization for Animal Health)
PAB	polyclonal antibody
PFGE	pulsed-field gel electrophoresis
PoC	point-of-care
ppb	parts per billion
PRRS	*porcine* reproduction and respiratory syndrome
PRV	pseudorabies virus
qPCR	quantitative (real-time) polymerase chain reaction
RCA	rolling circle amplification
RPA	recombinase polymerase amplification
RT	reverse transcription
RT-PCR	reverse transcription polymerase chain reaction
SDA	strand displacement amplification
SE	(relative) sensitivity
SERS	surface-enhanced Raman-spectroscopy
SP	(relative) specificity
SPR	surface plasmon resonance
STEC	Shiga toxin-producing *Escherichia coli*
TTR	time-to-result
VBNC	viable but non-culturable
UKAS	United Kingdom Accreditation Service
VOC	volatile organic compound
WHO	World Health Organization
xPOCT	multiplexed point-of-care testing
